# Income raises human well-being indefinitely, but age consistently slashes it

**DOI:** 10.1038/s41598-023-33235-7

**Published:** 2023-04-11

**Authors:** Chao Li, Shunsuke Managi

**Affiliations:** Urban Institute, Kyushu University, 744 Motooka, Nishi-ku, Fukuoka, 819-0395 Japan

**Keywords:** Quality of life, Health policy

## Abstract

The relationships among human well-being, income, and age have long been debated. The association between human well-being and income is believed to be U-shaped, although the reasons remain elusive. A recent study shows a turning point in the link between human well-being and income; that is, increased income does not always improve well-being. However, the mechanisms of the effects of income and age on human well-being are unknown. Here, we illustrate the total cumulative effects of income and age on evaluated well-being through all observed causal pathways based on a 1.6-million-observation global dataset and the structural causal model. This is the first study to investigate those casual relationships globally. We find that an increase in age always reduces evaluated well-being, and the adverse effects are aggravated with age. Furthermore, increased income continuously improves human well-being, but the impacts gradually become marginal with higher income. Our results demonstrate that physical health improvement in older people is the most effective way to intervene against the harmful effects of age on well-being. Moreover, increased income may dramatically enhance the well-being of people living close to the poverty line.

## Introduction

The relationships among human well-being, income, and age are inconclusive^1–6^. Whether and why there is a turning point in the relationship between income and well-being remains elusive^1,2^. One theory argues that increased income will eventually reach a ceiling at which human well-being does not further improve with income^2^. However, other evidence argues that no such turning point has been observed since both evaluated and hedonic human well-being continue to rise indefinitely with income^1,6^. Furthermore, the association between age and well-being has long been believed to be U-shaped^5,7^. Well-being hits a low point in middle age^5,7,8^. This phenomenon has consistently been observed in previous studies, but few clarify the reasons for this strange relationship^3,8,9^.

Studies have consistently shown that income is positively related to subjective well-being (SWB)^1,2,10,11^. Although the benefits of increased income for SWB gradually diminish, the impacts of income are positive overall^4,12^. When Kahneman and Deaton indicated that high income only improves evaluated well-being but not hedonic well-being^6^, this long-standing belief began to be questioned. Soon after, Jebb et al. concluded that the effect of income satiation on well-being exists globally according to an analysis of the Gallup World Poll (GWP) dataset^2^. An increase in income no longer poses a benefit for SWB when people’s primary needs are met^2^. The positive effects of increased income on SWB follow a pathway from the increase in income to greater fulfillment of needs to SWB improvement^12–15^. When fulfillment is not blocked or extra consumption is no longer desired, satiation occurs^2^. Even worse, negative impacts of higher income might emerge because high income is usually accompanied by high levels of responsibility and heavy workload and cannot easily be offset by fulfillment^16^. However, the opposition to satiation theory is also substantial. Killingsworth’s study showed that experienced well-being continues to rise with income even beyond the turning point identified in previous studies, using a long panel dataset including a large number of repeated, real-time observations^1^. Money helps people reduce suffering and increase enjoyment^10,11,17^. Moreover, people with a higher income feel more control over their own lives^1^. Sustainable education affects the attitude to income change due to the growth of economies because sustainable education is to equip people with the necessary understanding to help them to safeguard environmental, social, and economic well-being^18,19^. Moreover, sustainable education is an essential part of building a future civil society^20^. People with relatively higher income are apt to gain sustainable education. Therefore, an increase in income continuously improves well-being. The two sides of previous studies have one essential assumption in common: income partially and indirectly affects human well-being through other factors. Without mediators, the strength of the association between income and well-being declines. If the pathways of this relationship are identified, we could determine the mechanisms of the effects of increased income on well-being.

The relationship between well-being and age has been further investigated^3,5,8,9,21^. Numerous studies claim that the relationship between well-being and age is U-shaped^5,7,21^. Two explanations are widely cited: the selection explanation and the hedonic adaptation explanation. The selection explanation hypothesizes that people with high well-being tend to live longer^7,22^. The hedonic adaptation explanation suggests that people first feel the pain of impossible aspirations around midlife and begin to adapt to them^5,21^. These two explanations account for the U-shaped relationship based on the specific mediator. Physical health supports the selection explanation^23,24^. Happiness leads to better physical health, healthy people live longer, and surviving older adults enhance the means of human well-being. The hedonic adaptation explanation has a similar mechanism, but the mediator is mental health^5,25,26^. The pains of impossible aspirations are felt and then there is a gradual acceptance of reality^27,28^. Furthermore, evidence shows that low-income households are more likely to experience mental disorders in midlife^29^. Hence, income is a potential mediator of the relationship between human well-being and age. Aging is associated with gradually worsening health^30,31^, which might cause a reduction in income. Furthermore, people need time to obtain work experience and build social relationships, so income might peak in midlife. Therefore, the relationships among well-being, income, and age are complex. In this unified study, we examine the relationship networks.

Evaluated human well-being, represented by the Cantril ladder, is regarded as the measurement of SWB. In contrast with previous studies^1,6^, we examine experienced hedonic well-being to represent mental status of observations. First, in the GWP, personal health measurement mainly relies on these items. If we convert them into one explained variable, the effects of the mediator, health, will be obscured. Second, daily experience is a direct factor in mental health status^32,33^. In other words, although all the results are illustrated by evaluated well-being, hedonic well-being is also considered in the analysis processes. Additionally, the relationship between age and well-being is indirect^1,2^. Aging causes poor health and low income, which are attributable to decreased well-being. Put differently, without a change in other conditions, a marginal increase in age contributes to a lack of variation in human well-being. Hence, in the inference networks, age should not connect directly to well-being but should be linked by other variables’ pathways. In contrast, the effects of increased income on human well-being are both direct and indirect. Indirect effects include higher consumption or fulfilment of needs due to increased income^13^. Social comparison effects can reflect the direct effects of income^34,35^. Evidence shows that the effects of relative income are no weaker than the impact of absolute income^36^. Therefore, in the inference networks, income affects human well-being directly and indirectly.

## Materials and methods

### Materials

#### Survey information

We investigate the effects of age, income and other variables of interest on well-being through the GWP dataset for 18 years, from 2005 to 2022. The records were collected from over 2.4 million people from 168 countries or regions. This might be the largest worldwide dataset focusing on human well-being and has been widely used in previous studies^2,3^. The GWP survey typically samples at least 1000 individuals in every country and every wave. The information and explanations of the sampling and data collection methodology of the GWP are listed on Gallup’s website (https://www.gallup.com/178667/gallup-world-poll-work.aspx). In some countries, some questions of interest are not asked in some years. Additionally, if the respondents do not provide valid information for several essential questions, including life evaluation, age, and gender, these observations are removed from the final dataset for the analyses. The final dataset includes, approximately 1.6 million observations and covers 163 countries or regions from 2009 to 2022. Supplementary Materials Table S1 summarizes the sample sizes in each country and each survey wave.

#### Measurement of subjective well-being

Previous studies have shown that subjective well-being (SWB) objectively represents human well-being^4,37^. To measure SWB, three different approaches focusing on different aspects of well-being are widely considered and accepted^3^: life evaluation^4,37,38^, hedonic well-being^39^, and eudemonic well-being. The life evaluation method extracts well-being from people’s thoughts about the quality or goodness of their overall life^4,40^. In the Gallup survey, the 11-step Cantril ladder question is employed. Respondents are required to imagine a ladder with steps numbered from 0 at the bottom representing the worst possible life for them to 10 at the top representing the best possible life. Then, the respondents select the step of the ladder on which they feel that they currently stand. The number of the selected step is the respondent’s life evaluation. Because life evaluation is straightforward to understand and widely used in previous studies^4^, it is adopted as the explained variable in our study.

Unlike the life evaluation method, the hedonic well-being approach concentrates on daily experienced feelings and moods^7,41^. These feelings and moods generally involve positive and negative dimensions. Daily experienced feelings reflect human mental and physical health. Physical and mental health is one of the determining factors of individuals’ life evaluations^4,17,42^. Thus, in this study, daily experienced feelings and moods are the explanatory variables of life evaluation. In the Gallup survey, ten daily experienced feelings and moods are measured: feeling well-rested, feeling treated with respect, smiling or laughing, feeling something interest, enjoyment, physical pain, worry, sadness, stress, and anger. The respondents are asked whether they experienced the abovementioned feelings a lot during the previous day. The answers to these questions are all binary (i.e. yes or no). The respondents have the right to refuse to answer the questions or simply answer that they do not know. Considering rejections as missing data and then dropping the observations would decrease the data size dramatically. We employ a yes-is-yes strategy to process the dataset. In the yes-is-yes strategy, unless the respondents clearly answer “yes” to the questions, the answer should always be “no”. It must be noted that the yes-is-yes strategy is applied to all binary variable data processing except gender. Eudemonic well-being measures human well-being by self-judgments about the meaning and purpose of one’s life^40^. Due to a lack of well-investigated data in the GWP, eudemonic well-being is not considered in this study.

#### Other necessary variables

The fundamental analysis in this study stems from three core exogenous variables: age, gender, and the respondent’s country. Numerous previous studies show that the statistical relationship between age and well-being is a U-shape^3,5,7,8^ and that the well-being of females tends to be higher than that of males’^4,43^. However, the mechanisms of these associations are unclear. Thus, age and gender are taken into account in our analyses. Furthermore, the country is directly related to the respondents’ income since the income gaps between countries are significant. Therefore, the country variable must be considered.

The relationship between income and well-being remains inconclusive: some researchers suggest that increased income does not further improve well-being when income exceeds a certain level^2,6^, whereas others maintain that the effect of increased income is always positive^1^. The GWP dataset includes individual income data in international dollars, which facilitates the detection of the relationship. All waves of income data are converted into purchasing power parity based on the 2016 U.S. dollar. Therefore, the income data in the dataset are on a unified metric and comparable. Although it is unclear whether a turning point exists in the relationship between income and well-being, the logarithmic relationship is solidly supported^2,10,14^. We logarithmize annual income as follows:

1 $$LInc_{i} = \ln \;(Inc_{i} + 1)$$

where ${Inc}_{i}$ represents the individual annual income in international dollars of respondent $i$, and ${LInc}_{i}$ represents the natural logarithm of the income of respondent $i$.

An insufficient food index and insufficient shelter index are used in this study^10,13,15,44^. These two indexes assess whether people have the ability to meet basic needs for food and shelter. Respondents answer whether there have been several times in the past 12 months when they have been unable to buy sufficient food and provide adequate shelter to meet their or their families’ needs. To investigate people’s health, respondents are asked whether they have any health problems that prevent them from doing anything that people of the same age could normally do, which is known as disabilities due to health problems in this study. Other questions that evaluate personal health in the GWP were introduced in the hedonic well-being section, so we do not repeat them here. Living environments are associated with well-being, as indicated by previous studies^45–47^. Seven variables related to community basics are included in the analyses: whether people feel satisfied with public transportation, roads and highways, the quality of air, the quality of water, availability of housing, the educational system, and quality of healthcare. Evidence shows that community attachment is related to well-being^48^. Three community attachment-related variables are considered: whether the respondents are satisfied with the city, whether they are likely to move away from the city, and whether they recommend the city to their friends. Employment status is associated with well-being directly and indirectly. Therefore, employment status is also considered in this study.

### Methods

#### Relationships among variables

The relationship network is depicted using the directed acyclic graph (DAG) language^49,50^. To make the following parts understandable, we briefly introduce the terminology here. The DAG consists of variables and their relationship. All variables in the DAG are called nodes. If there is an association between two variables, we link them by an edge and call the edge a pathway. If the pathway represents a causal association, this pathway is directed from the reason variable to the result variable. The reason variables of a specific variable are called the parents of that variable, while the result variables are called children. In the DAG, the directed path is represented as an edge with an arrow. Three patterns of multivariable relationships appear in this study: chains, forks, and colliders^49,50^. There are three nodes in each DAG, A, B, and C. If A is a parent of B, and B is a parent of C, we say the pattern of A, B, and C is a chain. A and B are called the ancestors of C, while B and C are regarded as the descendants of A. Assuming no other associations exist in this DAG, if we condition node B, the patent association between A and C would disappear. If B is a parent of A and C, we say the pattern of A, B, and C is a fork. In this fork pattern without other associations, if we condition node B, the patent relationship between A and C would also vanish. Finally, if B is a child of A and C, we say the pattern of A, B, and C is a collider. In this collider pattern, A and C are statistically independent if there are no other correlations. When we condition B in this collider pattern, A and C would be conditionally related. The causal relationships are all based on either untestable assumptions or statistical tests. The untestable assumptions are only unable to be tested in this study due to a lack of variables, but they might be able to be proven in other studies.

We have several assumptions to build a relationship network of the variables of interest. The direct effect pathways from exogenous to endogenous variables are deemed valid even though there may not be solid statistical evidence to support them. Gender as an exogenous variable directly affects unemployment^51,52^ and income^52,53^. In some occupations, significant gender discrimination exists^54^. Evidence shows that the gender unemployment gap and the gender wage gap are still substantial in the U.S.^52^. Age, as another essential exogenous variable, directly impacts several endogenous variables, including unemployment^55^, income^56^, disabilities due to health problems^57,58^, physical pain, being treated with respect, smiling or laughing, enjoyment, interest, feeling well-rested, stress, anger, worry, and sadness^5^. The relationship between age and perceived external employability is significantly negative^55,59^. In several European countries, the mean income peaks in middle age^56^. With increased experience, individual income should rise, but aging also causes health problems that disturb working status and lead to a reduction in salary. In fact, as age increases, the probability of disabilities due to health problems also increases^57,58^. Evidence shows that nearly all daily experience items are associated with age^5^. Due to the differences in development among countries, the country in which respondents live has a direct impact on individual income. Moreover, the country was not decided by most of respondents. Hence, in this study, the country variable is also deemed exogenous.

Two variables, whether people feel satisfied with the educational system and air quality, are nontypical exogenous variables. Initially, we reasoned that income is a cause of satisfaction with the educational system because those with more resources can choose better schools. Additionally, age is a parent of income. Therefore, age should be an indirect cause of satisfaction with the educational system. In this case, the association between age and satisfaction with the educational system should decrease if income is conditioned because a potential pathway is blocked. However, the result is reversed; that is, the association increases. This result is consistent with only one pattern, a collider^49,50^: both satisfaction with the educational system and age are potential reasons for income. The true reason for high income is a better education system, which is also the reason for satisfaction with the educational system. Put differently, a causal association between income and satisfaction with the educational system does not exist. Since there are no reasonable parents in our dataset, such as investment in the local education system, the variable of satisfaction with the educational system is considered exogenous.

Satisfaction with air pollution is the other nontypical exogenous variable in this study. Initially, we suspect that income is a potential cause of satisfaction with air pollution since people with high income have more opportunities to choose where they live. However, the pattern of age, income and satisfaction with air pollution is a collider, age and satisfaction with air pollution are parents of income. In reality, high income also offsets the negative impacts of air pollution to improve people’s satisfaction with air quality^47,60^. After deeply investigating this relationship, we find no direct connection between income and satisfaction with air pollution due to the unobserved variable of air pollution density. Air pollution density is a confounder of income and satisfaction with air pollution. Highly polluted cities have more industries and factories, so their development level is normally better than that of other places, which is related to high salaries^61^. Low air pollution density causes satisfaction with air quality. A better development level is the cause of higher income, which is related to satisfaction with air quality. Because of the relationship between satisfaction with air quality and the development level, the statistical test among age, income and satisfaction with air pollution draw an incorrect conclusion. The relationships between satisfaction with air quality and disabilities due to health problems and physical pain are similar to the association between income and satisfaction with air quality. According to statistical tests, satisfaction with air quality is seemingly the reason for disabilities due to health problems and physical pain, but the real contributor is air pollution density. Since, in our dataset, there is no direct cause of satisfaction with air quality, satisfaction with air quality is also deemed an exogenous variable.

**Figure 1:**
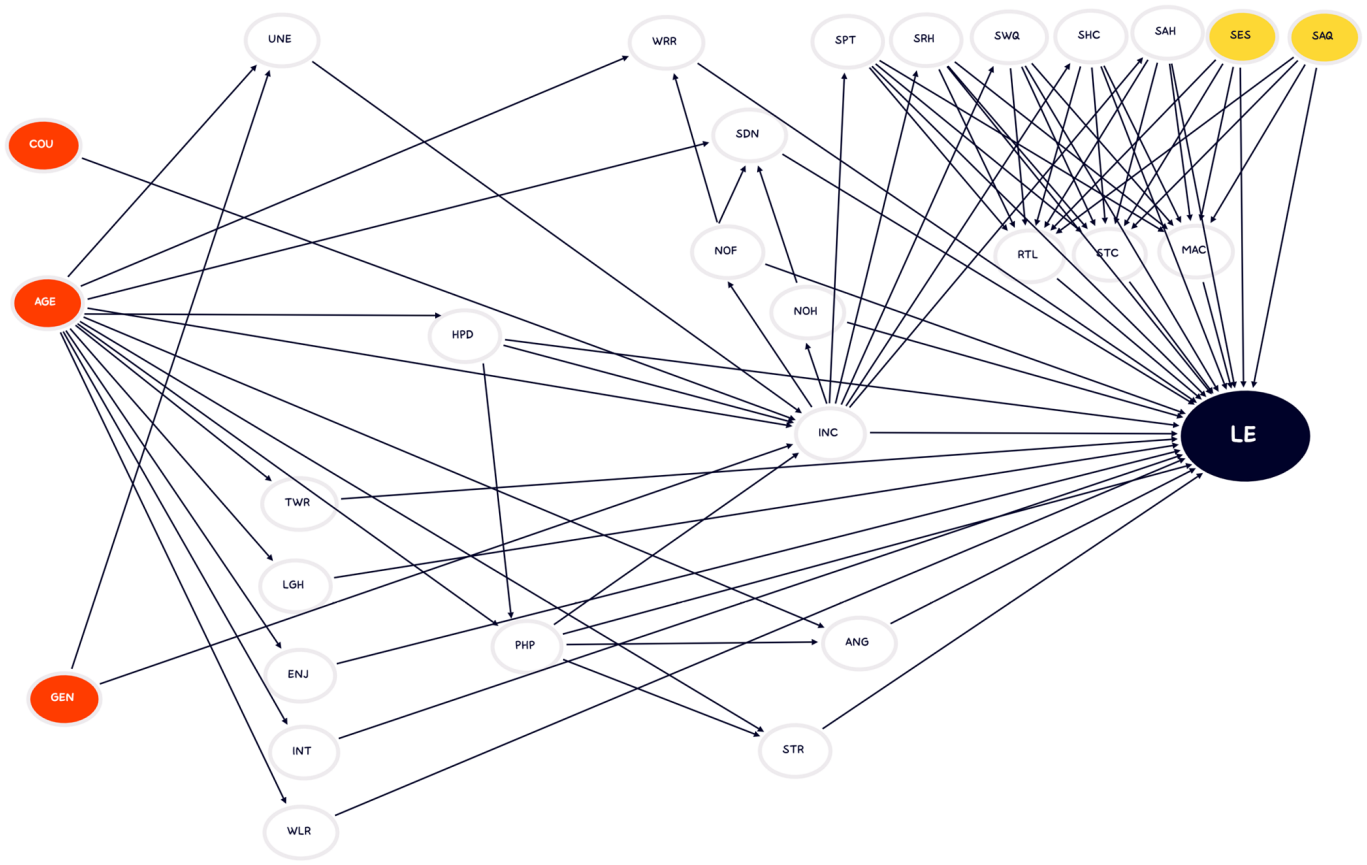
The DAG of the relationships among variables. ($UNE$, $TWR$, $LGH$, $ENJ$, $INT$, $WLR$, $STR$, $SDN$, $ANG$, $WRR$, $PHP$, $HPD$, $INC$, $NOF$, $NOH$, $SPT$, $SRH$, $SES$, $SAQ$, $SWQ$, $SHC$, $SAH$, $RTL$, $STC$, $MAC$, and $LE$ are unemployment, feeling treated with respect, smiling or laughing, enjoyment, feeling something interesting, feeling well-rested, stress, anger, worry, physical pain, disability due to health problem, logarithm of individual income, Insufficient food index, insufficient shelter index, satisfaction with public transportation, roads and highways, educational system, quality of air, quality of water, quality of healthcare, and availability of housing, satisfaction with the city, recommending the city to their friends, moving away from the city and life evaluation).

The endogenous variables and the edges are illustrated and supported. If the parents of a variable are all exogenous, they are not mentioned in this section to avoid repetition. The variable that has fewer parents except exogenous variables is demonstrated first. According to a statistical test, disability due to health problems is a cause of physical pain. The statistical correlation coefficient between age and physical pain is 15.39% (p value < 0.1%), but after conditioning disability due to health problems, the conditional correlation coefficient decreases to 7.22% (p value < 0.1%). The correlation between age and physical pain is broken by blocking the causal pathway. Previous studies show that disability leads to more stress, although the causes of disability are not mentioned^62^. Physical pain is a factor of stress that is inferred based on another statistical test. The statistical correlation coefficient between disability due to health problems and stress is 8.37% (p value < 0.1%), but after conditioning for physical pain, the conditional correlation coefficient decreases to 1.78% (p value < 0.1%). Apparently, physical pain impedes the pathway from disability due to health problems and stress. Moreover, physical pain is also judged as a parent of anger based on a similar pathway test. The association coefficient between disability due to health problems and anger is 7.43% (p value < 0.1%), while the conditional correlation on physical pain decreases to 1.87% (p value < 0.1%). Income is ascribable to unemployment, disability due to health problems, and physical pain. The pathway from age to income could be partially hindered by conditioning unemployment. The reasoning for disability due to health problems is complicated due to an unobserved variable, physical health. The conditional association between age and disability due to health problems when controlling income is higher than the direct association. It can be inferred that both disabilities due to health problems and age are potential causes of income because the pattern of these three variables is a collider. However, when we compare the conditional association between age and income controlling for disability due to health problems, we obtain another collider pattern showing that income and age are parents of disability due to health problems. In fact, physical health, the ignored confounder of income and disability due to health problems causes this strange phenomenon. In the GWP survey, the aim of questions about disability due to health problems and physical pain is to imply physical health. Therefore, we accept that disability due to health problems is a cause of income. Since the situations of disability due to health problems and physical pain are the same, physical pain is also deemed a factor of income. Insufficient food and shelter indexes are attributable to income because the respondents are directly asked whether they have enough money to buy food and provide adequate shelter. An insufficient food situation is a cause of worry. Statistical test results show that after controlling for the insufficient food index, the conditional association between income and worry shrinks to 1.87% (p value < 0.1%), while the direct relationship is 7.43% (p value < 0.1%). Income is a factor of satisfaction with public transportation, roads and highways, quality of water, availability of housing, and quality of healthcare. The pathways from age to income to these variables are confirmed. Moreover, an insufficient shelter situation is a parent of satisfaction with the availability of housing since the connection between income and satisfaction with the availability of housing may be partially blocked by the conditioning of the insufficient shelter index. In this study, we assume that seven community-based variables are concrete reasons for the three community attachment-related variables. Finally, we assume that all the abovementioned variables except gender, age, and country are directly linked to the result of life evaluation. The DAG of the relationships among variables is demonstrated in Fig. 1.

Previous studies have probed the relationships between income and hedonic well-being^1,2,10,15^. Income might be a cause of the daily experience items. We examine the pathways from income to the daily experience items to evaluated well-being, but nothing is observed. Income is not a parent of the daily experience items, at least not, in this DAG. The questions to obtain the daily experience data lead to this situation. The questions asking “Did you experience the following feelings during a lot of the day yesterday?” have the disadvantage of representing a longer period, although this reduces the error possibility due to recall. The effect of income is difficult to mirror in a random single daily experience. Hence, in this research, the pathways from income to daily experience items to evaluated well-being are not observed and considered.

#### Structural causal model

In our structural causal model (SCM), 23 endogenous variables, two nontypical exogenous variables, and one final explained variable are built into the equations. Hence, the whole SCM has 26 equations, which are listed as follows:

2.1 $$UNE = f_{UNE} \;\left( {AGE, \;GEN,\;U_{UNE} } \right)$$

2.2 $$TWR = f_{TWR} \;\left( {AGE,\; U_{TWR} } \right)$$

2.3 $$LGH = f_{LGH} \;\left( {AGE, \;U_{LGH} } \right)$$

2.4 $$ENJ = f_{ENJ} \;\left( {AGE, \;U_{ENJ} } \right)$$

2.5 $$INT = f_{INT} \;\left( {AGE, \;U_{INT} } \right)$$

2.6 $$WLR = f_{WLR} \;\left( {AGE, \;U_{WLR} } \right)$$

2.7 $$STR = f_{STR} \;\left( {AGE, \;PHP,\;U_{STR} } \right)$$

2.8 $$SDN = f_{SDN} \;\left( {AGE,\;NOF,\;NOH\,U_{SDN} } \right)$$

2.9 $$ANG = f_{ANG} \;\left( {AGE,\;PHP, \;U_{ANG} } \right)$$

2.10 $$WRR = f_{WRR} \;\left( {AGE,\;NOH, \;U_{WRR} } \right)$$

2.11 $$PHP = f_{PHP} \;\left( {AGE,\;HPD, \;U_{PHP} } \right)$$

2.12 $$HPD = f_{HPD} \;\left( {AGE, \;U_{HPD} } \right)$$

2.13 $$INC = g_{INC} \;\left( {UNE,\;COU,\;AGE,\;GEN,\;HPD,\;PHP, \;U_{INC} } \right)$$

2.14 $$NOF = f_{NOF} \;\left( {INC, \;U_{NOF} } \right)$$

2.15 $$NOH = f_{NOH} \;\left( {INC,\; U_{NOH} } \right)$$

2.16 $$SPT = f_{SPT} \;\left( {INC, \;U_{SPT} } \right)$$

2.17 $$SRH = f_{SRH} \;\left( {INC, \;U_{SRH} } \right)$$

2.18 $$SES = f_{SES} \;\left( {U_{SES} } \right)$$

2.19 $$SAQ = f_{SAQ} \;\left( {U_{SAQ} } \right)$$

2.20 $$SWQ = f_{SWQ} \;\left( {INC, \;U_{SWQ} } \right)$$

2.21 $$SHC = f_{SHC} \;\left( {INC, \;U_{SHC} } \right)$$

2.22 $$SAH = f_{SAH} \;\left( {INC, \;U_{SAH} } \right)$$

2.23 $$RTL = f_{RTL} \;\left( {SPT,\;SRH,\;SES,\;SAQ,\;SWQ,\;SHC,\;SAH,\; U_{RTL} } \right)$$

2.24 $$STC = f_{STC} \;\left( {SPT,\;SRH,\;SES,\;SAQ,\;SWQ,\;SHC,\;SAH, \;U_{STC} } \right)$$

2.25 $$MAC = f_{MAC} \;\left( {SPT,\;SRH,\;SES,\;SAQ,\;SWQ,\;SHC,\;SAH, \;U_{MAC} } \right)$$

2.26 $$\begin{aligned} LE & = g_{LE} (UNE, \;TWR,\; LGH, \;ENJ, \;INT,\; WLR, \;STR,\;SDN,\;ANG,\;WRR, \\ & \quad PHP, \;HPD, \;INC,\;NOF,\;NOH, \;SPT, \;SRH, \;SES, \;SAQ, \;SWQ,\; SHC, \;SAH, \\ & \quad RTL, \;STC, \;MAC, \;U_{LE} ) \\ \end{aligned}$$

where $UNE$, $TWR$, $LGH$, $ENJ$, $INT$, $WLR$, $STR$, $SDN$, $ANG$, $WRR$, $PHP$, $HPD$, $INC$, $NOF$, $NOH$, $SPT$, $SRH$, $SES$, $SAQ$, $SWQ$, $SHC$, $SAH$, $RTL$, $STC$, $MAC$, and $LE$ represent unemployment, feeling treated with respect, smiling or laughing, enjoyment, interest, feeling well-rested, stress, anger, worry, physical pain, disability due to health problem, logarithm of individual income, insufficient food index, insufficient shelter index, satisfaction with public transportation, roads and highways, educational system, quality of air, quality of water, quality of healthcare, and availability of housing, satisfaction with the city, recommending the city to friends, moving away from the city and life evaluation, respectively; $U_{y}$ represents the unobserved causes of the variable $y$; $f_{y}$ and $g_{y}$ represent the function taking the variable $y$ as the explained variable. Because most explained variables except age and life evaluation are binary, their functions should be a logistic regression, written as $f_{y}$. For age and life evaluation, the functions are an ordinary least squares (OLS) regression, written as $g_{y}$.

#### Cumulative effect in SCM

The cumulative effect of a certain variable of interest on a specific explained variable in this SCM is rarely stationary because most functions are a logistic regression, $f_{y}$. The logistic regression, $f_{y}$, between the variable of interest, $x$, and the probability of the explained variable, $y$, is expressed as follows:

3 $$\ln \left( {\frac{{P_{yi} }}{{1 - P_{yi} }}} \right) = ax_{i} + b_{i}$$

where $P_{yi}$ represents the probability of $y$ of observation $i$, $x_{i}$ represents the value of the variable of interest of observation $i$, $a$ represents the estimated slope of $x$, and $b_{i}$ represents the intercept of observation $i$. In Eq. (2), some functions have several explanatory variables. Equation (2.1) is a case in point. Gender and age are the causes of unemployment, but we need to hold the value of gender constant as an intercept if we only focus on the effect of age on unemployment. This is the reason for the variability of $b_{i}$. Based on Eq. (3), the derivative function of $P_{yi}$ can be written as follows:

4 $$\frac{{dP_{yi} }}{{dx_{i} }} = \frac{{ae^{{ax_{i} + b_{i} }} }}{{\left( {1 + e^{{ax_{i} + b_{i} }} } \right)^{2} }}$$

The effect of *x*_*i*_ on *P*_*yi*_ is affected by the value of *x*_*i*_ itself and the value of other causes of *y*, as shown in Eq. (4). The responses of all *f*_*y*_ functions are the probability of *y*, rather than the value of *y*. To make the calculation effective, we directly accept several assumptions. First, in logistic regression, the change in the probability of the explained variable can be conveyed to the next-level regression as the real change in this variable, which can be written as follows:

5 $$\frac{{dy_{i} }}{{dx_{i} }} = \left\{ {\begin{array}{*{20}c} {\frac{{dP_{yi} }}{{dx_{i} }}, } & {\quad if\; the\; connection \;function \;is \;logistic\; regression} \\ {\frac{{dy_{i} }}{{dx_{i} }}, } & {\quad if\; the \;connection \;function\; is \;OLS\; regression} \\ \end{array} } \right.$$

Second, all the derivatives of each variable in each regression based on Eq. (4) are regarded as stationary. Based on these two assumptions, the cumulative effect of a certain pathway can be calculated as follows:

6 $$\frac{{dv_{ni} }}{{dv_{li} }} = \prod \frac{{dv_{{\left( {m + 1} \right)i}} }}{{dv_{mi} }}$$

where $\frac{{dv_{ni} }}{{dv_{li} }}$ is the cumulative effect following the pathway from variable $l$ to variable $n$, and $\frac{{dv_{{\left( {m + 1} \right)i}} }}{{dv_{mi} }}$ shows the derivatives of the $m$ level regression. For instance, the cumulative effect following the pathway from age to physical pain to stress can be computed as follows:

7 $$\frac{{dSTR_{i} }}{{dAGE_{i} }} = \frac{{dPHP_{i} }}{{dAGE_{i} }} \times \frac{{dSTR_{i} }}{{dPHP_{i} }}$$

where $\frac{{dSTR_{i} }}{{dAGE_{i} }}$ represents the cumulative effect following the pathway of observation $i$, $\frac{{dPHP_{i} }}{{dAGE_{i} }}$ represents the effects of age on the physical pain of observation $i$, and $\frac{{dSTR_{i} }}{{dPHP_{i} }}$ represents the effects of physical pain on the stress of observation $i$. The total accumulative effects from variable $l$ to variable $n$ are the sum of the cumulative effects following all the observed pathways as follows:

8 $$TAE_{li}^{n} = \mathop \sum \limits_{p = 1}^{P} \frac{{dv_{pni} }}{{dv_{pli} }}$$

where $TAE_{li}^{n}$ represents the total cumulative effect of the variable $l$ on the variable $n$ of the observation $i$, $\frac{{dv_{pni} }}{{dv_{pli} }}$ represents the cumulative effect the following pathway $p$, and $P$ is the total number of the observed pathways from the variable $l$ to the variable $n$. Here, we must emphasize that $TAE_{l}^{n}$ is the marginal change in the variable $n$ when the variable $l$ increases by 1 unit, which can be regarded as slopes or derivatives. In this study, we concentrate on three associations: the relationships between age and income, age and SWB, and income and SWB.

## Results and discussion

Unlike previous studies, our SCM grasps the mechanisms of age and income’s effects on human well-being according to the casual chains. Based on the estimated coefficients following Eq. (2) (listed in Supplementary Materials Table S2) and the dataset, the total cumulative effects of age on the natural logarithm of individual income, age on life evaluation, and the natural logarithm of individual income on the life evaluation of each observation are estimated. It must be emphasized that most parts of the SCM are fitted by logistic regressions, so the relationships between variables are non-linear. As shown in Eq. (4), both the variables of interest and other explanatory variables influence the derivatives.

### Total cumulative effects of age on life evaluation

**Figure 2:**
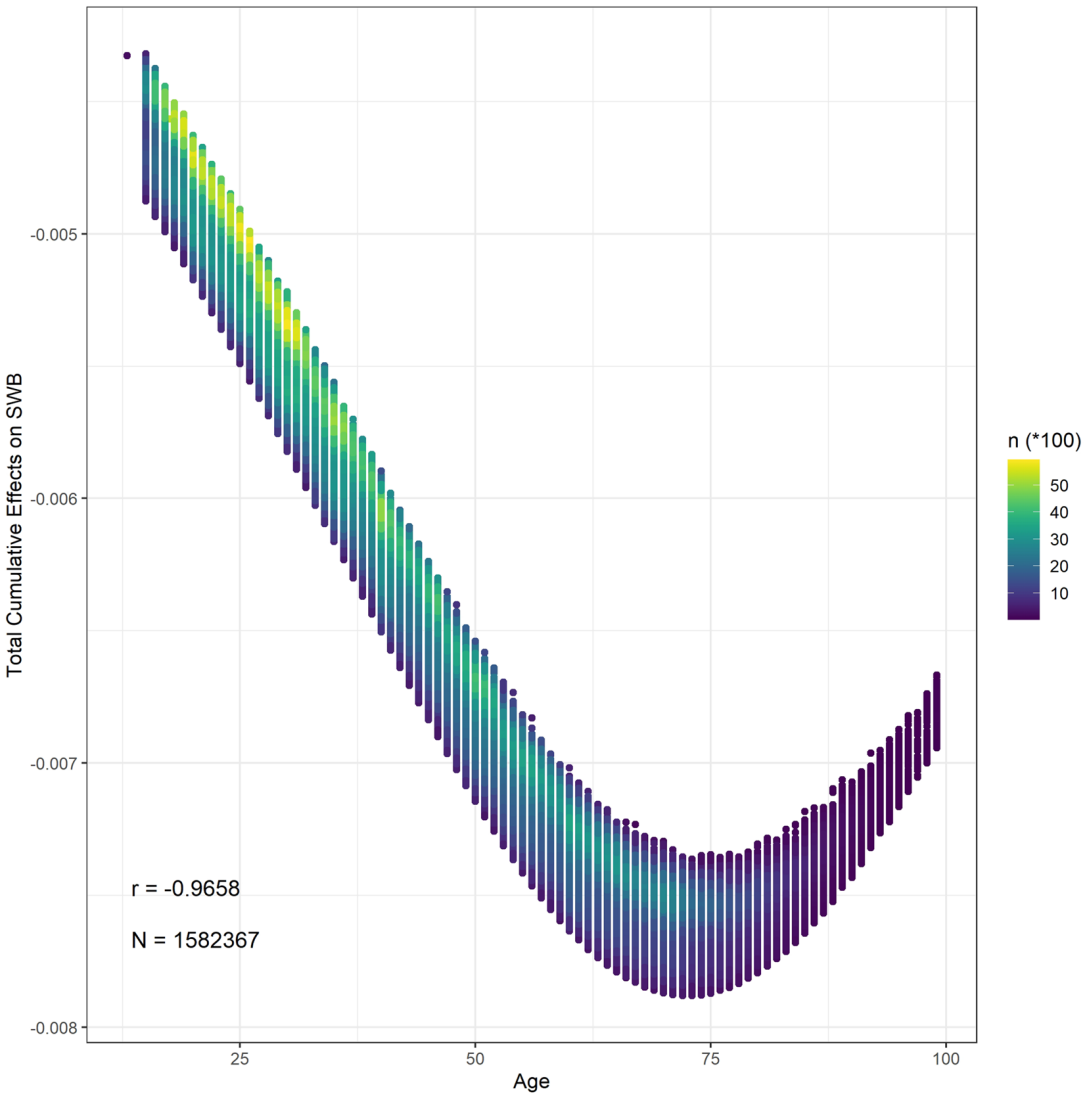
The distribution between the total cumulative effects of age on SWB and age.

The total cumulative effects of age on life evaluation span from $- \;7.880 \times 10^{ - 3}$ to $- \;4.320 \times 10^{ - 3}$ with a mean of $- \;6.114 \times 10^{ - 3}$ (95% confidence inference: $- \;6.115 \times 10^{ - 3}$ to $- \;6.112 \times 10^{ - 3}$). Figure 2 demonstrates the scatter plot of age’s total cumulative effects on life evaluation and age. The logistic regression mathematically and statistically causes the nonlinearity of the relationship. Because the total cumulative effects of age on life evaluation can be regarded as derivatives, the negative value means that increased age is always negatively associated with life evaluation. The absolute values of the total cumulative effects decrease when age is over 70. This phenomenon might be ascribed to selection bias. The number of observations over the age of 70 is relatively lower than other age groups. Furthermore, happy people are more likely to have greater longevity^5,7,63^. The SCM provides insights into the mechanisms of the relationship between age and well-being. According to Eq. (2), the contributions of age to each explanatory variable in Eq. (2.26) can be estimated and demonstrated. Table 1 summarizes the cumulative effects of each explanatory variable in Eq. (2.26). The cumulative effects of negative factors in Eq. (2.26) on life evaluation are almost positive, while the cumulative effects of positive aspects are nearly all adverse. Increased age leads to a reduction in life evaluation following almost any observed pathways in the DAG. Put another way, increased age worsens most of the factors that affect life evaluation.

**Table 1:** The cumulative effects of age on each variable affecting life evaluation.

Statistic	N	Mean	St. dev.	Min	1st Quantile	3rd Quantile	Max
INC(log)	1,582,367	− 0.001	0.000	− 0.001	− 0.001	− 0.001	− 0.001
SPT	1,582,367	− 0.00004	0.00001	− 0.0001	− 0.0001	− 0.00003	− 0.00002
SRH	1,582,367	− 0.0001	0.00002	− 0.0001	− 0.0001	− 0.0001	− 0.00002
SAQ	1,582,367	0.000	0.000	0	0	0	0
SWQ	1,582,367	− 0.0001	0.00002	− 0.0001	− 0.0001	− 0.00004	− 0.00001
SAH	1,582,367	− 0.0001	0.00002	− 0.0001	− 0.0001	− 0.0001	− 0.00002
SES	1,582,367	0.000	0.000	0	0	0	0
SHC	1,582,367	− 0.0001	0.00002	− 0.0001	− 0.0001	− 0.0001	− 0.00002
HPD	1,582,367	0.007	0.002	0.003	0.005	0.009	0.010
WLR	1,582,367	− 0.001	0.00002	− 0.001	− 0.001	− 0.001	− 0.001
PHP	1,582,367	0.004	0.001	0.002	0.003	0.004	0.005
WRR	1,582,367	0.002	0.0002	0.002	0.002	0.002	0.002
SDN	1,582,367	0.002	0.0005	0.001	0.002	0.002	0.003
TWR	1,582,367	0.001	0.00004	0.0005	0.001	0.001	0.001
LGH	1,582,367	− 0.003	0.0003	− 0.003	− 0.003	− 0.003	− 0.002
INT	1,582,367	− 0.003	0.00004	− 0.003	− 0.003	− 0.003	− 0.003
ENJ	1,582,367	− 0.002	0.0001	− 0.002	− 0.002	− 0.002	− 0.002
STR	1,582,367	− 0.001	0.0002	− 0.001	− 0.001	− 0.001	− 0.0003
ANG	1,582,367	− 0.001	0.0004	− 0.003	− 0.002	− 0.001	− 0.001
NOF	1,582,367	0.0002	0.0001	0.00000	0.0001	0.0002	0.0003
NOH	1,582,367	0.0001	0.00003	0.00001	0.0001	0.0001	0.0002
STC	1,582,367	− 0.00002	0.00001	− 0.0001	− 0.00003	− 0.00001	− 0.00000
MAC	1,582,367	0.00001	0.00000	0.00000	0.00000	0.00001	0.00001
RTL	1,582,367	− 0.00001	0.00000	− 0.00001	− 0.00001	− 0.00000	− 0.00000
UNE	1,582,367	− 0.001	0.0004	− 0.002	− 0.002	− 0.001	− 0.0005

Why is the impact of increased age not in line with previous studies? Previous studies have focused on the statistical association between age and SWB. Adding a quadratic term in the regression might improve the goodness of fit, and the coefficients of the term might be significant, especially in cross-sectional research^5,8^. Previous studies mainly find that the statistical relationship between SWB and age is a U-shape^3,7,21,64^. However, some reject the U-shaped relation^17,65^, although non-U-shaped relationships are not as widely accepted. Practically, the reasons for the U-shaped relationship are elusive. The U-shaped relationship between age and SWB is superficial, obscuring the real causes. Aging provokes a variety of changes within and between individuals, including physical and mental health, attitudes toward life, and social relationships^3,5,30,31,33^. These changes impact well-being, positively or negatively, and the effects might vary with age. The combination of positive and negative effects is reflected as a U-shaped relationship. Hedonic adaption can explain this phenomenon after adding the quadratic term of age; however, most studies have not considered adaptation^7,8^. According to hedonic adaptation theory, unhappiness due to impossible aspirations appears around midlife, and the self-regulatory mechanism emerges later^21^. Our study adopts hedonic adaptation theory and follows previous studies in accepting the possibility of a nonlinear relationship between age and SWB. We examine each potential pathway of negative or positive effects from age to SWB. We assume that items of hedonic well-being, or daily experience, are the direct factors of SWB. In those ten daily experience-related items, increased age aggravates the possibility of negative emotions and feelings but reduces the opportunity for positive emotions with the exception of the feeling of being treated with respect, which increases with increasing age. However, additional respect cannot offset the harmful effects of other feelings. Hedonic well-being is negatively implicated in age in this way. Furthermore, aging gives rise to a decline in body function, which is the largest part of the adverse effects^30,33^. Acceptance of impossible aspirations might occur as time passes^5^, whereas physical health worsens. The most likely reason for the difference between our study and previous studies is that potential pathways from age to SWB are not observed. Therefore, in terms of the current network, age reduces SWB.

### Total cumulative effects of income on life evaluation

**Figure 3:**
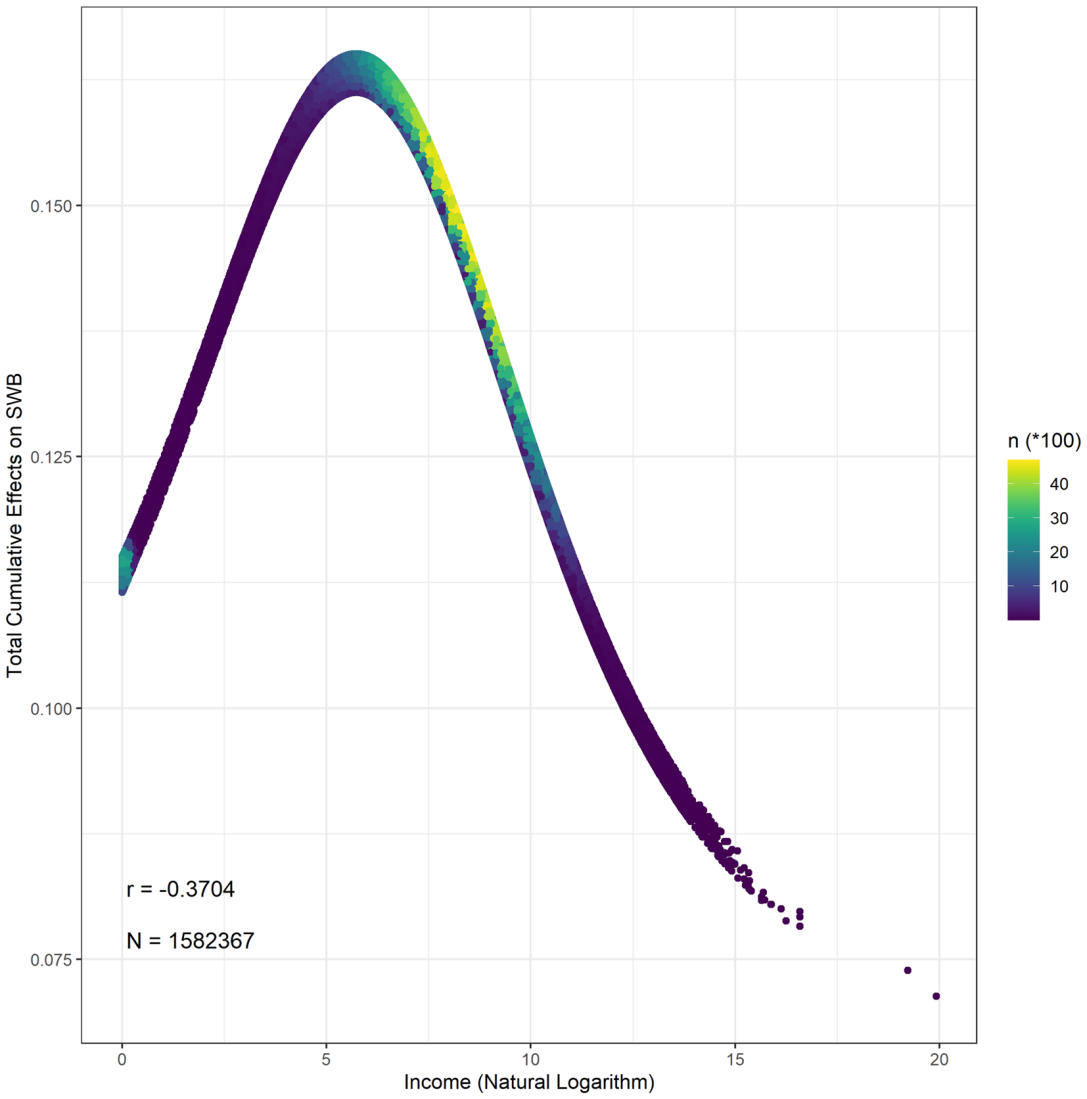
The distribution between the total cumulative effects of income on SWB.

The total cumulative effects of the natural logarithm of individual income on the life evaluation mean is 0.146 (95% CI 0.146–0.146), scaling from 0.071 to 0.165. As shown in Fig. 3, the relationship between the total accumulated effects and income is close to a bell shape. The total cumulative effects of the natural logarithm of income peak at approximately 6.5, which is 665 USD/year and close to the World Bank’s global poverty line, 1.90 USD/day. In other words, increased income contributes most to human well-being before reaching the poverty line. Although the contributions of increased income to well-being decrease, they are always positive. Previous statistical evidence worldwide shows that well-being does not increase further after arriving at a certain turning point^2^, while others illustrate that the increased income is associated with higher well-being without a threshold based on a survey in the U.S.^1^. The direct and indirect impacts of the natural logarithm of individual income on life evaluation are positive (shown in Supplementary Materials Table S2). Specifically, the increase in the natural logarithm of individual income following each pathway improves well-being. If the turning point exists, there must be at least one pathway with a negative cumulative impact. Furthermore, the total negative impacts offset the positive impacts. Then, increased income no longer improves well-being. However, in terms of the current evidence, we do not find a valid pathway that offers the negative cumulative effects on the relationship between income and life evaluation (Table 2). To conclude, increased income causes an improvement in life evaluation without any turning point, consistent with the previous evidence^1^.

**Table 2:** The cumulative effects of income on each variable affecting life evaluation.

Statistic	N	Mean	St. dev.	Min	1st Quantile	3rd Quantile	Max
INC(log)	1,582,367	0.000	0.000	0	0	0	0
SPT	1,582,367	0.020	0.0004	0.014	0.020	0.020	0.021
SRH	1,582,367	0.033	0.001	0.017	0.033	0.034	0.034
SAQ	1,582,367	0.000	0.000	0	0	0	0
SWQ	1,582,367	0.029	0.002	0.010	0.028	0.031	0.035
SAH	1,582,367	0.008	0.001	0.005	0.008	0.009	0.010
SES	1,582,367	0.000	0.000	0	0	0	0
SHC	1,582,367	0.035	0.001	0.015	0.034	0.035	0.036
HPD	1,582,367	0.000	0.000	0	0	0	0
WLR	1,582,367	0.000	0.000	0	0	0	0
PHP	1,582,367	0.000	0.000	0	0	0	0
WRR	1,582,367	0.000	0.000	0	0	0	0
SDN	1,582,367	0.000	0.000	0	0	0	0
TWR	1,582,367	0.000	0.000	0	0	0	0
LGH	1,582,367	0.000	0.000	0	0	0	0
INT	1,582,367	0.000	0.000	0	0	0	0
ENJ	1,582,367	0.000	0.000	0	0	0	0
STR	1,582,367	0.000	0.000	0	0	0	0
ANG	1,582,367	0.000	0.000	0	0	0	0
NOF	1,582,367	− 0.079	0.017	− 0.099	− 0.094	− 0.068	− 0.002
NOH	1,582,367	− 0.044	0.008	− 0.061	− 0.050	− 0.038	− 0.004
STC	1,582,367	0.009	0.004	0.002	0.005	0.013	0.015
MAC	1,582,367	− 0.003	0.001	− 0.004	− 0.003	− 0.002	− 0.001
RTL	1,582,367	0.003	0.001	0.002	0.003	0.004	0.004
UNE	1,582,367	0.000	0.000	0	0	0	0

Increased income consistently elevates SWB, although the effects gradually temper as income increases. The results support studies that focus on the lack of income satiation. In our study, all observed pathways from income to SWB convey positive effects. Thus, the cumulative effects of increased income on SWB are beneficial. The nonlinearity of the relationship also involves the combination of several direct and indirect effects attributable to increased income. On the one hand, increased income provides more consumption, including materials and services, to improve well-being^13^. On the other hand, high demands, such as heavy workload, large amounts of responsibility, and long worktimes, combined with high incomes reduce human well-being^16^. Furthermore, due to social comparison, income directly influences human well-being. Previous studies have examined the relationship between hedonic well-being and income^1,2,6^. Jebb et al. claim that there is also a turning point in the relationship between hedonic well-being and income^2^. However, in our study, we do not find a direct connection between the items of hedonic well-being and income based on nearly the same dataset used in Jebb et al.’s research. The counterintuitive situation is triggered by the question used in the GWP, which required respondents to recall their experience of the previous day. The effect of individual annual income might be mirrored by changes in the frequency of daily experience for a relatively long time. The association between hedonic well-being and income mentioned in Jebb et al.’s research might be caused by confounders. For example, age is a direct factor in both income and daily experience items. Additionally, there are unobserved confounders supporting the statistical association between hedonic well-being and income.

### Total cumulative effects of age on income

**Figure 4:**
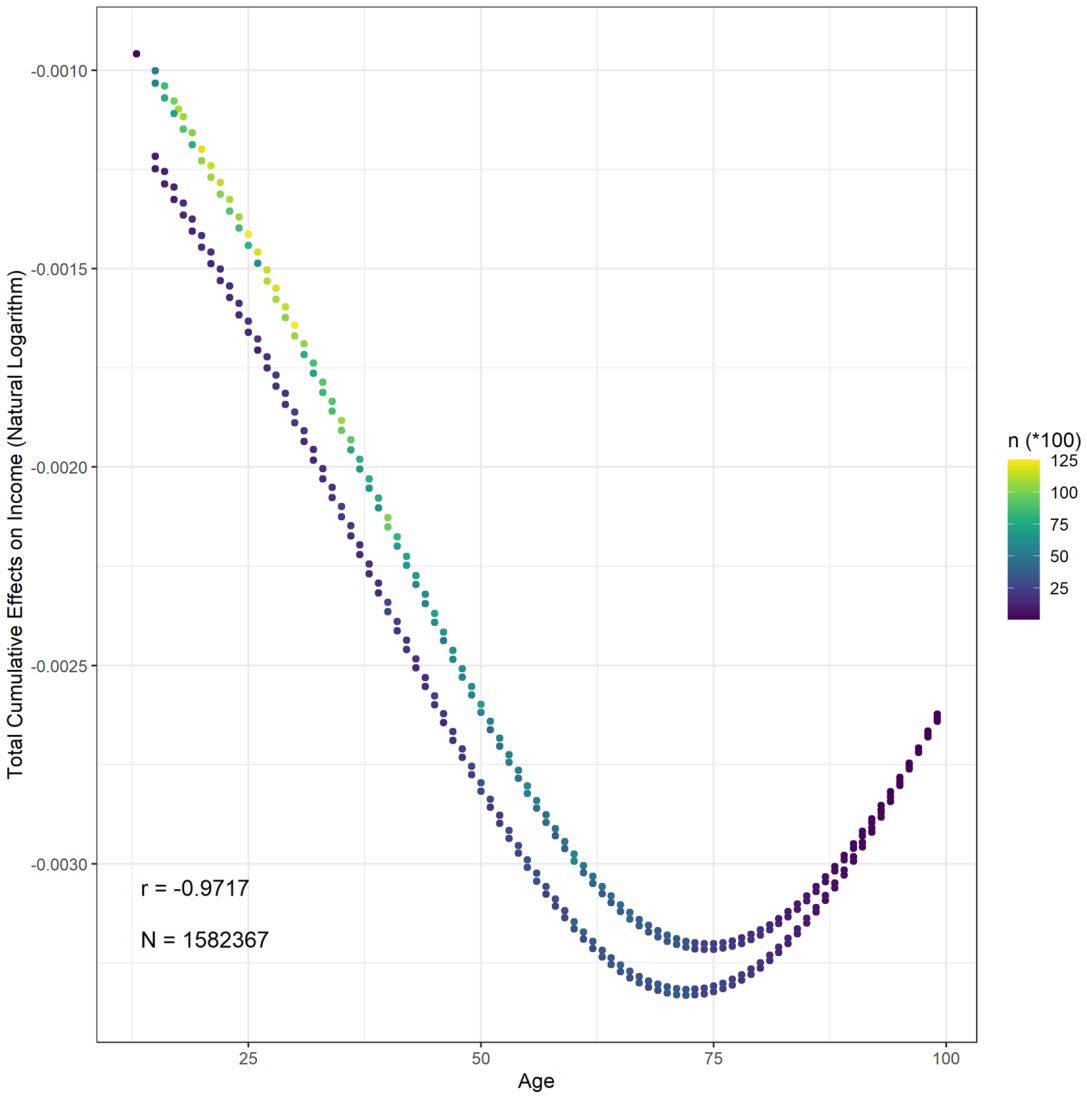
The distribution between the total cumulative effects of age on income.

The total cumulative effects of age on the natural logarithm of individual income range from $- \;3.332 \times 10^{ - 3}$ to $- \;9.583 \times 10^{ - 4}$ with a mean of $- \;2.158 \times 10^{ - 3}$ (95% CI $- \;2.159 \times 10^{ - 3}$ to $- \;2.156 \times 10^{ - 3}$). Figure 4 plots the relationship between the total cumulative effects of age on life evaluation. In the domain of our study, the function is monotonically decreasing since the total cumulative effects are always negative. In plain language, the increased age always causes a potential income reduction, and the effects are more substantial among older people. Table 3 summarizes the cumulative effects of age on each endogenous variable affecting income. It must be underscored that the total cumulative effects might change in a different SCM. The main reason for this change is that new pathways are considered. For example, according to the total cumulative effects of age on income, older people are more likely to obtain a lower income, which opposes the real world situation. Many uncomputable pathways cause this opposition. Work experience is a case in point. Experienced workers are older but more expensive due to higher efficiency. Furthermore, older people might have more investments because they use their savings to improve their income. Although there is space for improvement, the GWP, which is one of the most comprehensive surveys, provides the ability to investigate the nexus between income and age compared with previous studies.

**Table 3:** The Cumulative Effects of Age on Each Variable Affecting Income.

Statistic	N	Mean	St. Dev	Min	1st Quantile	3rd Quantile	Max
INC(log)	1,582,367	0.000	0.000	0	0	0	0
SPT	1,582,367	0.000	0.000	0	0	0	0
SRH	1,582,367	0.000	0.000	0	0	0	0
SAQ	1,582,367	0.000	0.000	0	0	0	0
SWQ	1,582,367	0.000	0.000	0	0	0	0
SAH	1,582,367	0.000	0.000	0	0	0	0
SES	1,582,367	0.000	0.000	0	0	0	0
SHC	1,582,367	0.000	0.000	0	0	0	0
HPD	1,582,367	0.007	0.002	0.003	0.005	0.009	0.010
WLR	1,582,367	0.000	0.000	0	0	0	0
PHP	1,582,367	0.004	0.001	0.002	0.003	0.004	0.005
WRR	1,582,367	0.000	0.000	0	0	0	0
SDN	1,582,367	0.000	0.000	0	0	0	0
TWR	1,582,367	0.000	0.000	0	0	0	0
LGH	1,582,367	0.000	0.000	0	0	0	0
INT	1,582,367	0.000	0.000	0	0	0	0
ENJ	1,582,367	0.000	0.000	0	0	0	0
STR	1,582,367	0.000	0.000	0	0	0	0
ANG	1,582,367	0.000	0.000	0	0	0	0
NOF	1,582,367	0.000	0.000	0	0	0	0
NOH	1,582,367	0.000	0.000	0	0	0	0
STC	1,582,367	0.000	0.000	0	0	0	0
MAC	1,582,367	0.000	0.000	0	0	0	0
RTL	1,582,367	0.000	0.000	0	0	0	0
UNE	1,582,367	− 0.001	0.0004	− 0.002	− 0.002	− 0.001	− 0.0005

### Country-level average total cumulative effects

**Figure 5:**
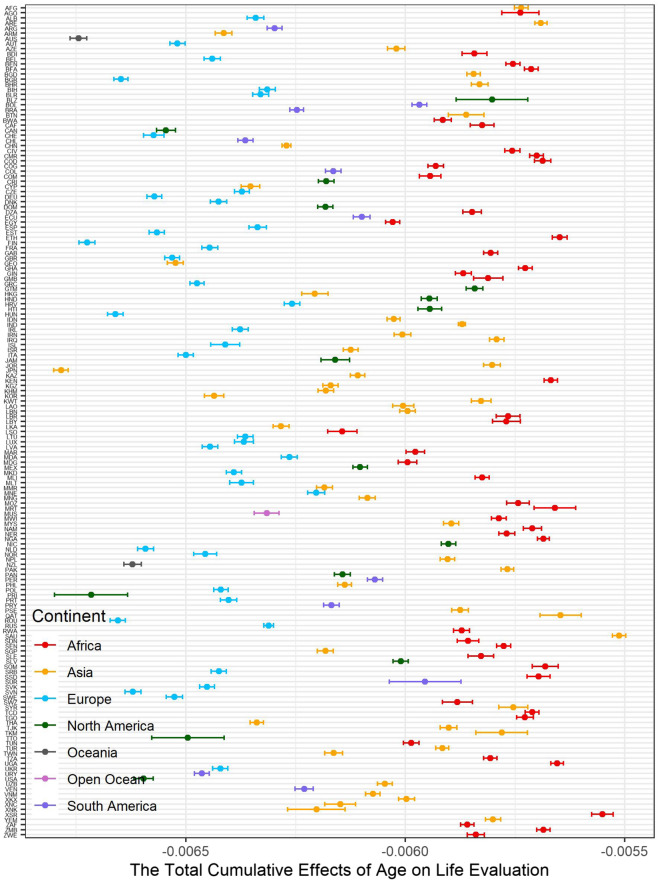
The summary of total cumulative effects of age on life evaluation by country.

**Figure 6:**
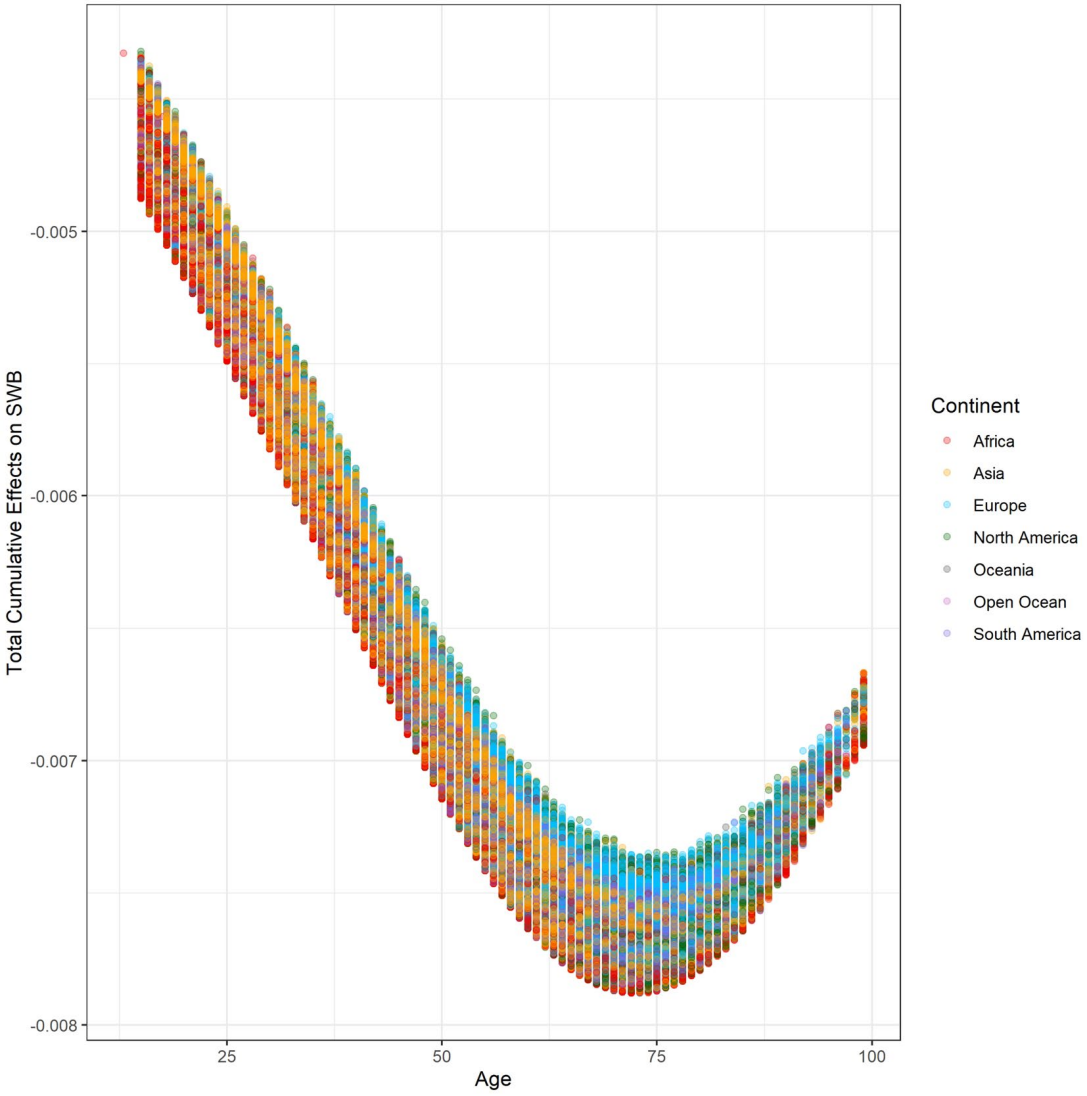
Distribution between the total cumulative effects of age on SWB and age by continent.

**Figure 7:**
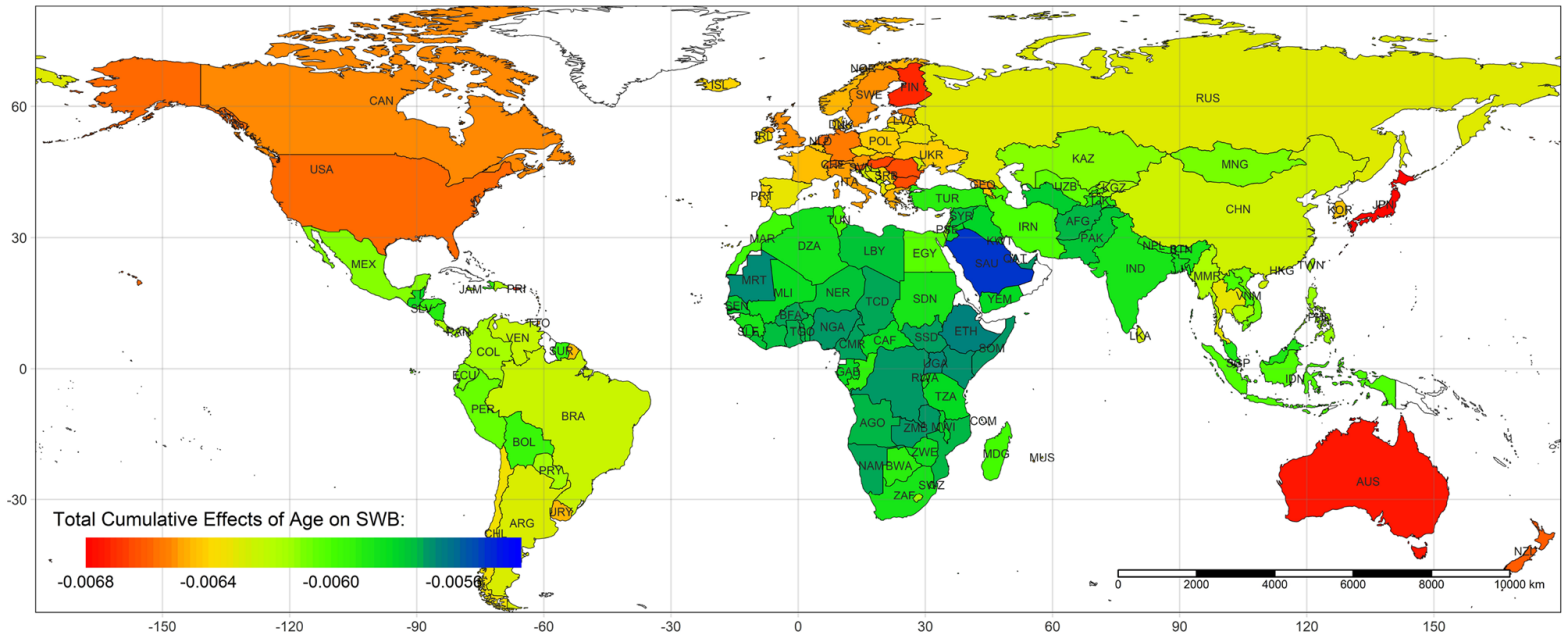
Map of the country-level average total cumulative effects of age on SWB.

The total cumulative effects of age on life evaluation are summarized by country (Fig. 5). We find that the absolute values of the average total cumulative effects of age on life evaluation in the Oceanic, European, and North American countries are larger than in other continents. We recolored Fig. 2 by continent (Fig. 6). At the same age, the negative effects of age on life evaluation are apparently more minor in Europeans than in Asians and Africans. The reason for the strongest negative effects is aging in developed countries is because longer life expectancy is also associated with higher average age^17,30^. Therefore, unsurprisingly, the minimum of the average total cumulative effects appears in Japan, which has the most difficult aging situation^66^. The lives of older people in developing countries, especially in Africa, is more difficult. The labor market, poor health care system, and welfare system in developing countries might worsen the situation. The average total cumulative effects by country show global variation (Fig. 7). The negative effects of increasing age are more significant in developed countries than in developing countries.

**Figure 8:**
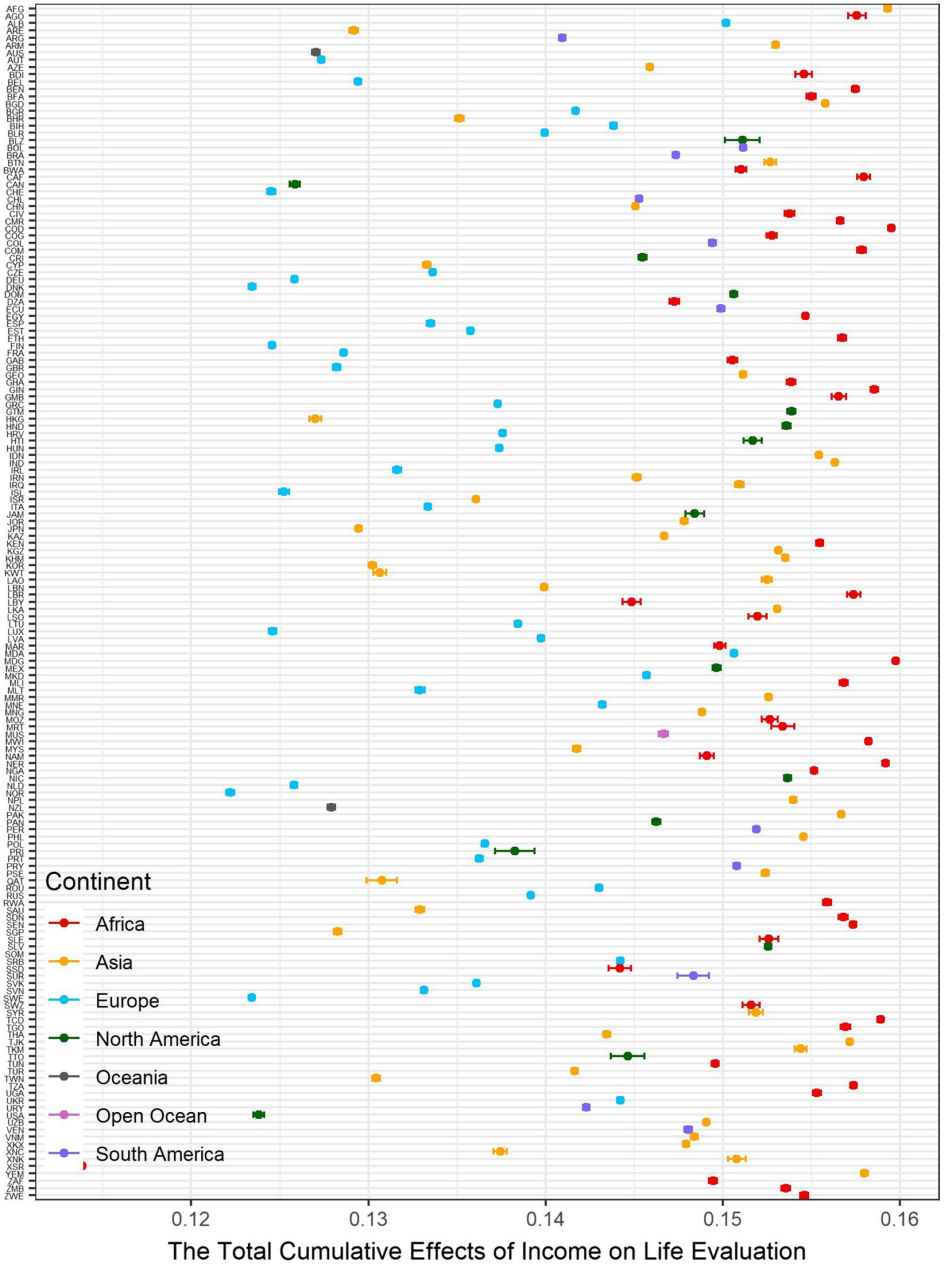
The summary of total cumulative effects of income on life evaluation by country.

**Figure 9:**
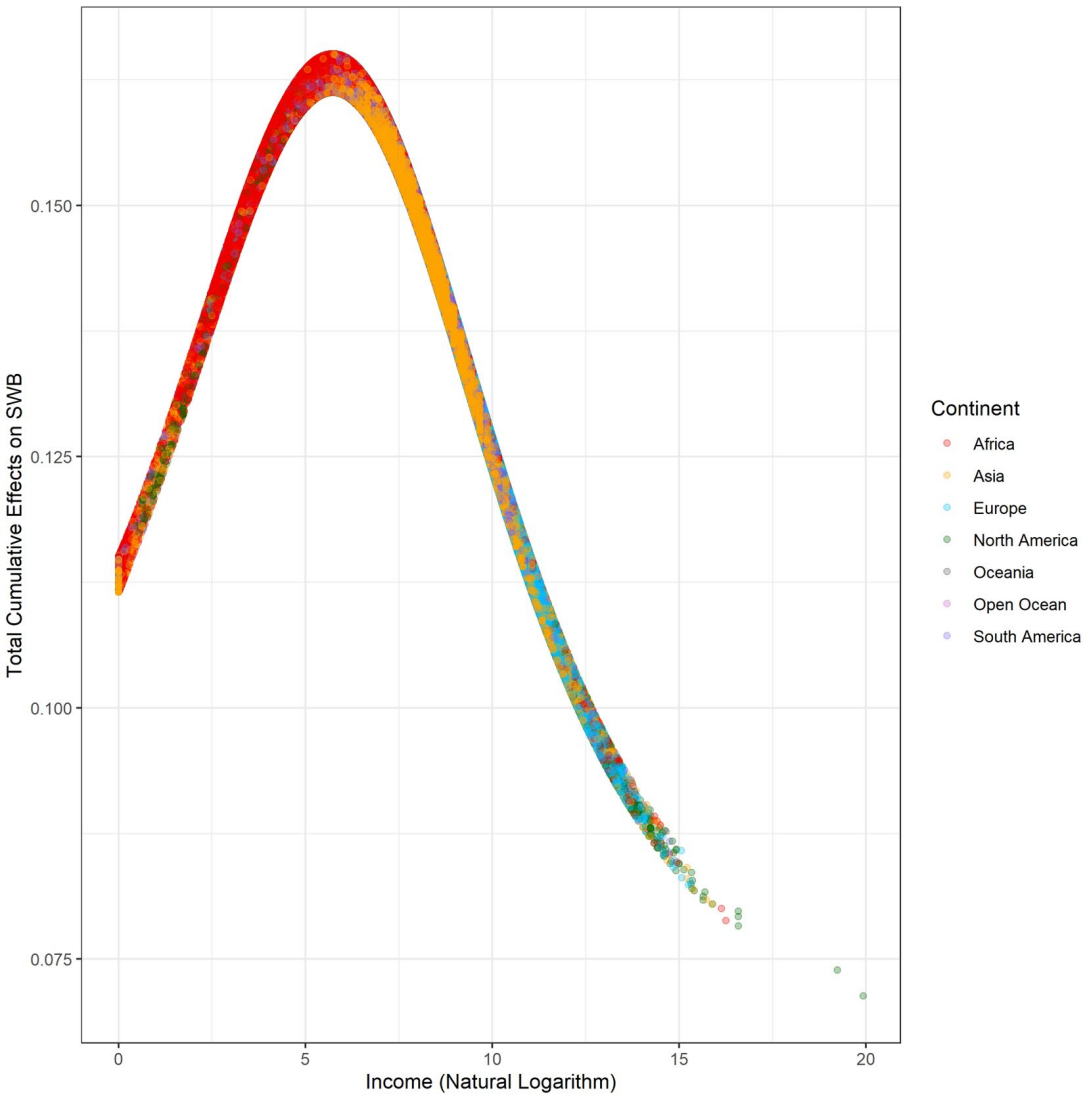
Distribution between the total cumulative effects of income on SWB and income by continent.

**Figure 10:**
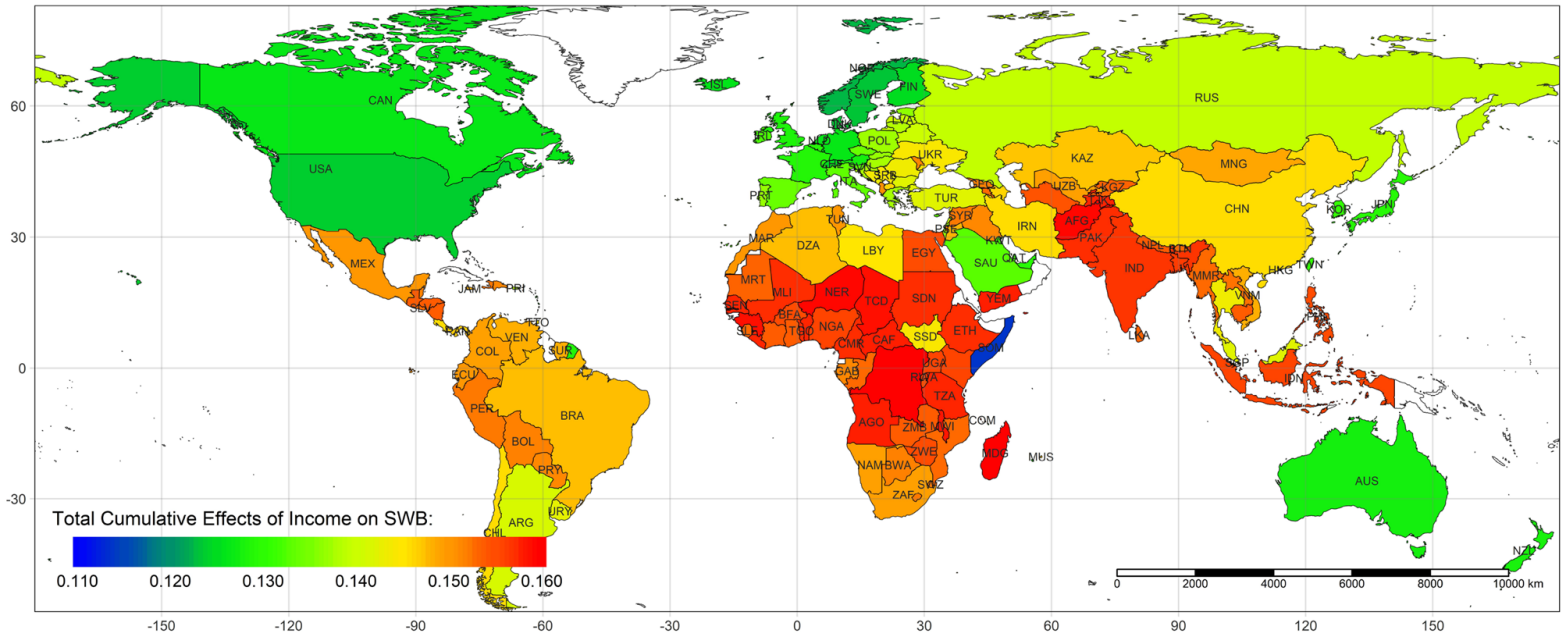
Map of country-level average total cumulative effects of income on SWB.

**Figure 11:**
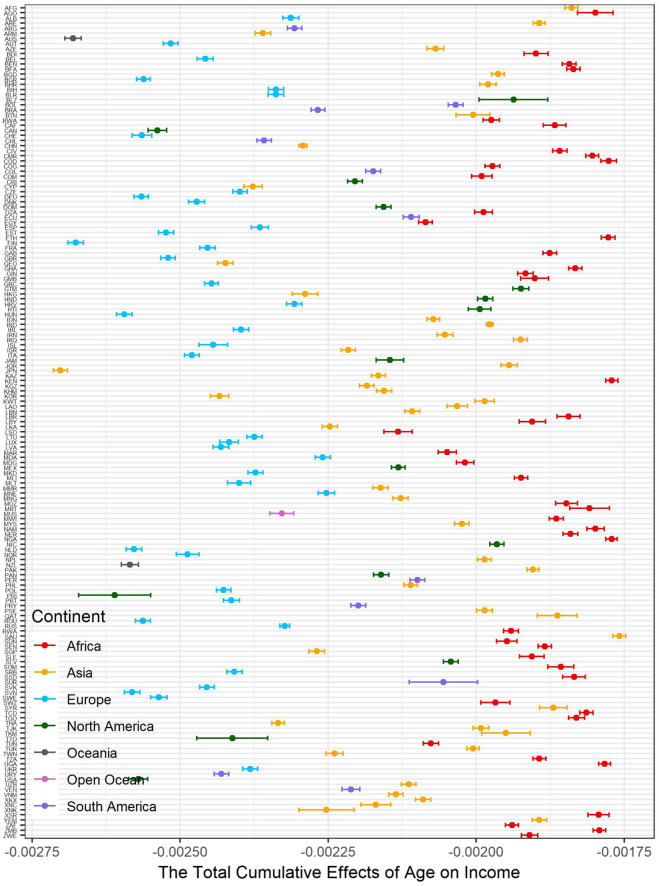
The summary of total cumulative effects of age on income by country.

**Figure 12:**
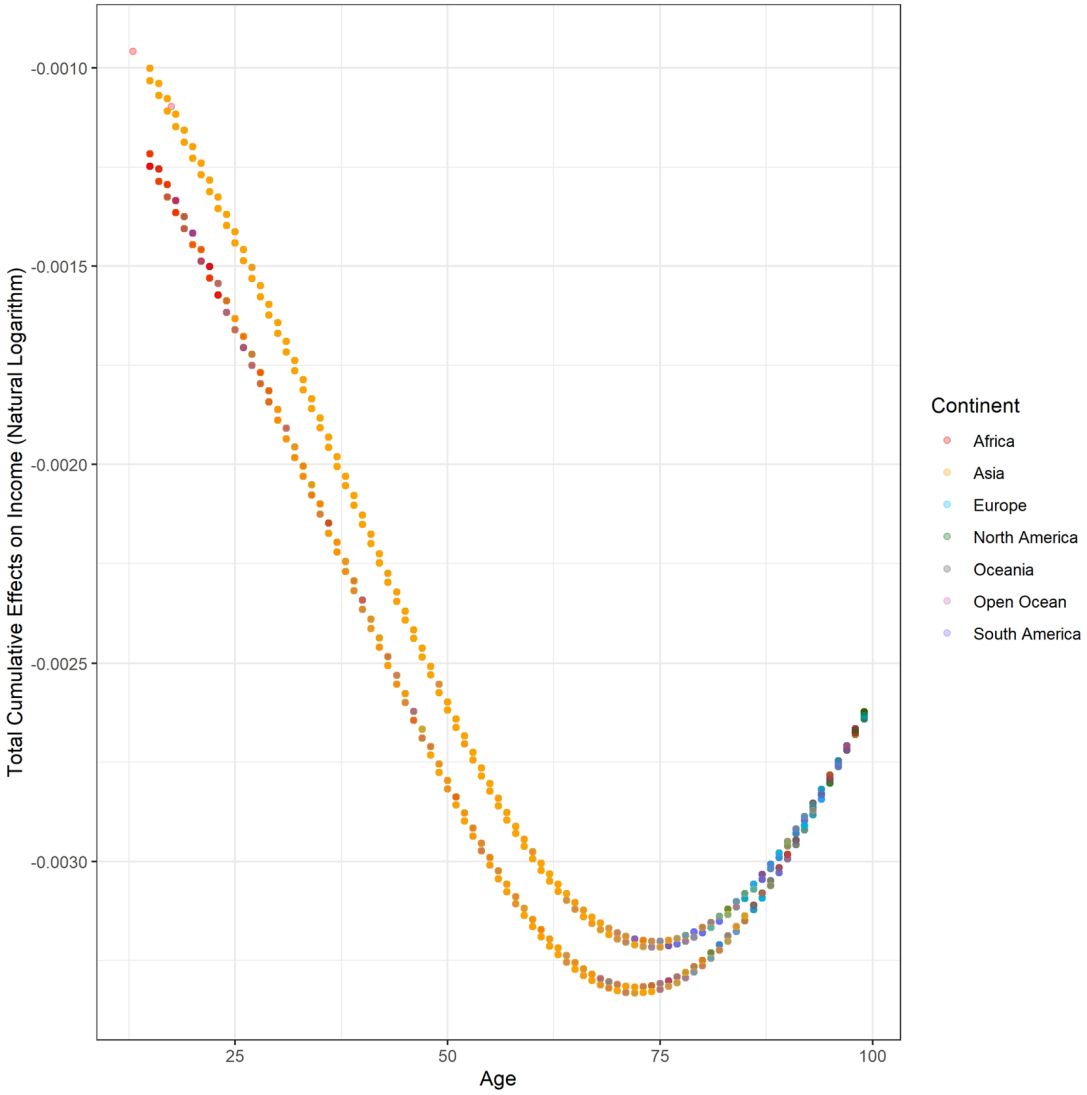
Distribution between the total cumulative effects of age on income and age by continent.

Figure 8 illustrates the country-level average total cumulative effects of income on life evaluation. Due to poverty, the positive effects of increased income are maximized in Africa. Figure 9 recolors the distribution between the total cumulative effects and income by continent. Almost all African and Asian observations are located in the higher total cumulative effects. The income in these countries is relatively lower than that in developed countries. Among these countries, the poverty of Somalia (SOM) is beyond imagination. The maximum individual income in Somalia is approximately 2.5 USD/year. In this awful situation, even a dramatic income increase cannot reverse the conditions due to pervasive poverty. Additionally, people in rich countries have more opportunities to obtain sustainable education, which could reduce the impacts of income change^19^, while poor countries should be more sensitive due to a lack of sustainable education. Sustainable education facilitates development and paves the way to future civil society^20^. Figure 10 demonstrates the spatial variation of the country-level average total cumulative effects of income on SWB. Figure 11 demonstrates the country-level average total cumulative effects of age on income. The situation of the effects of age on income is similar to the effects on life evaluation. Aging societies or countries with longer life expectation have the most negative average values. Figure 12 is a recolored version of Fig. 4, but the variation in each age is not discernable because only four pathways were detected in our study. Most observations overlap in Fig. 12. Too few observed causal variables are the reason for the overlap in Fig. 12.

The overall gap between countries in population age structure and the level of development causes the difference in country-level average effects of age and income on SWB. The level of development shapes the population age structure and determines a country’s role in the global industrial chain. In other words, the most common jobs between countries and regions are significantly different, and the basic demands are not similar. The average effects show spatial variability. Even at the same age, the adverse effects of age on SWB in Africa are larger than those in Europe. This phenomenon can be explained by the fact that jobs in Africa mainly concentrate on manual labor, where young people clearly have a more competitive advantage. Moreover, with different incomes, the aims of consumption vary. Theoretically, the fulfillment of needs improves well-being^14,15^. According to Maslow’s hierarchy of needs, needs are classified into five strata, from physiological needs to self-actualization^15,67^. Typically, lower-level needs rely on money more than higher-level needs. Put another way, increased income has a relatively weaker relationship with improved well-being when people already have high income. Therefore, the effect of a 1-unit increase in the natural logarithm peaks around the World Bank’s poverty line. The fulfillment of the basic needs of food and shelter is easiest.

### Limitations and future studies

Although we employ one of the largest worldwide datasets to investigate the relationships between age and SWB, income and SWB, and age and income, some improvements can be made if more data can be obtained. First, the causal relationship between age and income could be more deeply probed if variables, such as detailed educational background, work experience, among others, were available. In this study, the numbers of investigated pathways and mediators in the relationships are relatively limited. Second, although the GWP spans a long time, the observations in each wave are sampled randomly rather than surveying the same individuals for several years. Currently, we cannot eliminate the time-fixed effects within individuals to calibrate the relationship of interest. Third, because most variables in the analysis are binary, the connection function should be a logistic regression. However, logistic regression cannot perfectly convey the effects. In future studies, more effective mathematical tools should be created and applied to investigate the causal effects. Furthermore, panel datasets are strongly desired. Finally, more potential pathways from age or income to well-being should be examined to make the pattern more comprehensive.

## Conclusion

This is the first study to investigate the causal relationships among human well-being, income, and age using a global dataset. Intervention for gradually deteriorating health in an aging society is the most effective way to maintain or improve human well-being. While increasing age is not inherently detrimental to human well-being, it can lead to various physical and mental health issues that ultimately harm overall well-being. Additionally, the effectiveness of an increase in income is typically most pronounced when individuals initially have low incomes. Although a turning point in the relationship between well-being and income, such as 60,000 to 75,000 USD/year, regarding life evaluation was not detected, the contribution of increased income to life evaluation beyond these amounts is marginal. Increased income always leads to human well-being improvement because its effects on the direct and indirect factors are always positive in most pathways observed in this study.

## Supplementary Information

Supplementary Tables.

## Data Availability

The datasets used and/or analysed during the current study available from the corresponding author on reasonable request.
