# Supplementary material for: Income raises human well-being indefinitely, but age consistently slashes it
Source: Sci Rep. 2023 Apr 11;13:5905. doi: 10.1038/s41598-023-33235-7 (PMC10090099; doi:10.1038/s41598-023-33235-7)
Supplement: Supplementary file 1 — Supplementary Tables. [file 41598_2023_33235_MOESM1_ESM.docx]

Supplementary Materials:

Income raises human well-being indefinitely, but age consistently slashes it

# Authors

Chao Li^1^, Shunsuke Managi*^1^

# Affiliations

1 Urban Institute, Kyushu University, Japan

* Correspondent to: Shunsuke Managi, managi@doc.kyushu-u.ac.jp, Kyushu University 744 Motooka, Nishi-ku, Fukuoka 819-0395 Japan

# Table

| **Table S1: Observation Summary** | | | | | | | | | | | | | | |
| --- | --- | --- | --- | --- | --- | --- | --- | --- | --- | --- | --- | --- | --- | --- |
| Country Code ISO3 | 2009 | 2010 | 2011 | 2012 | 2013 | 2014 | 2015 | 2016 | 2017 | 2018 | 2019 | 2020 | 2021 | 2022 |
| AFG | 999 | 1000 | 1000 | 994 | 0 | 942 | 997 | 996 | 967 | 968 | 1120 | 0 | 998 | 0 |
| AGO | 0 | 0 | 0 | 0 | 985 | 972 | 0 | 0 | 0 | 0 | 0 | 0 | 0 | 0 |
| ALB | 0 | 993 | 1005 | 1029 | 1027 | 981 | 991 | 982 | 987 | 976 | 1069 | 986 | 980 | 0 |
| ARE | 0 | 1030 | 2015 | 0 | 0 | 0 | 2877 | 1833 | 0 | 0 | 0 | 989 | 935 | 0 |
| ARG | 997 | 992 | 998 | 997 | 1000 | 998 | 998 | 996 | 995 | 996 | 1056 | 1000 | 1000 | 0 |
| ARM | 997 | 997 | 984 | 990 | 993 | 990 | 990 | 991 | 988 | 984 | 1064 | 0 | 970 | 0 |
| AUS | 0 | 997 | 1000 | 0 | 994 | 980 | 979 | 977 | 0 | 0 | 0 | 970 | 962 | 0 |
| AUT | 0 | 1964 | 989 | 993 | 993 | 999 | 998 | 0 | 0 | 0 | 1021 | 998 | 995 | 0 |
| AZE | 0 | 0 | 990 | 988 | 968 | 978 | 991 | 980 | 987 | 989 | 1070 | 0 | 0 | 0 |
| BDI | 1000 | 0 | 1000 | 0 | 0 | 995 | 0 | 0 | 0 | 907 | 0 | 0 | 0 | 0 |
| BEL | 0 | 977 | 987 | 984 | 982 | 1000 | 1036 | 0 | 0 | 0 | 1025 | 1001 | 504 | 495 |
| BEN | 0 | 0 | 982 | 1000 | 998 | 996 | 986 | 954 | 910 | 943 | 990 | 1030 | 985 | 0 |
| BFA | 0 | 998 | 1000 | 1000 | 1005 | 997 | 993 | 961 | 960 | 918 | 919 | 0 | 1953 | 0 |
| BGD | 1000 | 1000 | 1000 | 977 | 989 | 0 | 984 | 992 | 976 | 992 | 2979 | 973 | 0 | 963 |
| BGR | 0 | 1956 | 1001 | 1000 | 991 | 998 | 998 | 993 | 994 | 993 | 1059 | 995 | 996 | 0 |
| BHR | 0 | 1022 | 1975 | 0 | 997 | 0 | 1991 | 996 | 0 | 0 | 0 | 0 | 0 | 0 |
| BIH | 0 | 1000 | 1008 | 1004 | 1006 | 995 | 993 | 997 | 992 | 999 | 1073 | 990 | 985 | 0 |
| BLR | 1031 | 991 | 966 | 1012 | 1007 | 1021 | 1017 | 1024 | 1048 | 1044 | 1097 | 0 | 0 | 0 |
| BLZ | 0 | 0 | 0 | 0 | 0 | 501 | 0 | 0 | 0 | 0 | 0 | 0 | 0 | 0 |
| BOL | 986 | 971 | 979 | 942 | 987 | 980 | 983 | 975 | 979 | 986 | 0 | 1981 | 999 | 0 |
| BRA | 1023 | 1036 | 1038 | 0 | 1997 | 999 | 998 | 983 | 971 | 971 | 2925 | 997 | 1000 | 0 |
| BTN | 0 | 0 | 0 | 0 | 997 | 0 | 1011 | 0 | 0 | 0 | 0 | 0 | 0 | 0 |
| BWA | 0 | 0 | 999 | 1000 | 1000 | 996 | 995 | 962 | 981 | 972 | 1036 | 0 | 0 | 0 |
| CAF | 0 | 998 | 999 | 0 | 0 | 0 | 0 | 990 | 959 | 0 | 0 | 0 | 0 | 0 |
| CAN | 987 | 988 | 679 | 0 | 574 | 1002 | 0 | 516 | 0 | 0 | 0 | 962 | 970 | 0 |
| CHE | 990 | 0 | 0 | 993 | 0 | 997 | 498 | 0 | 0 | 0 | 0 | 994 | 997 | 0 |
| CHL | 1008 | 1007 | 1007 | 1001 | 998 | 1019 | 1025 | 1001 | 1036 | 996 | 0 | 2056 | 999 | 0 |
| CHN | 3807 | 3426 | 4135 | 4206 | 4206 | 0 | 0 | 0 | 3931 | 3536 | 3577 | 3302 | 0 | 0 |
| CIV | 0 | 0 | 0 | 0 | 1006 | 999 | 989 | 969 | 955 | 941 | 980 | 998 | 978 | 0 |
| CMR | 0 | 1197 | 997 | 999 | 997 | 995 | 970 | 973 | 945 | 929 | 976 | 992 | 985 | 0 |
| COD | 999 | 0 | 988 | 999 | 998 | 1000 | 996 | 996 | 972 | 0 | 0 | 0 | 0 | 0 |
| COG | 0 | 0 | 998 | 499 | 988 | 978 | 987 | 974 | 915 | 905 | 1047 | 944 | 953 | 0 |
| COL | 995 | 996 | 992 | 994 | 997 | 990 | 998 | 981 | 982 | 981 | 972 | 999 | 995 | 0 |
| COM | 0 | 998 | 1999 | 999 | 0 | 0 | 0 | 0 | 0 | 958 | 948 | 0 | 0 | 0 |
| CRI | 993 | 1001 | 992 | 995 | 996 | 998 | 991 | 980 | 989 | 986 | 993 | 0 | 1975 | 0 |
| CYP | 491 | 991 | 989 | 492 | 489 | 976 | 1011 | 0 | 0 | 0 | 1031 | 986 | 1002 | 0 |
| CZE | 0 | 2036 | 996 | 996 | 998 | 1004 | 999 | 997 | 995 | 982 | 0 | 995 | 991 | 0 |
| DEU | 988 | 995 | 2553 | 999 | 741 | 1000 | 999 | 0 | 0 | 0 | 1010 | 994 | 996 | 0 |
| DNK | 998 | 996 | 1000 | 996 | 751 | 996 | 1002 | 0 | 0 | 0 | 1024 | 996 | 1009 | 0 |
| DOM | 990 | 990 | 997 | 989 | 990 | 986 | 979 | 985 | 977 | 974 | 1055 | 998 | 974 | 0 |
| DZA | 0 | 0 | 2000 | 0 | 0 | 0 | 0 | 0 | 1010 | 990 | 1090 | 1011 | 992 | 0 |
| ECU | 998 | 998 | 0 | 999 | 0 | 997 | 1000 | 998 | 991 | 985 | 991 | 989 | 995 | 0 |
| EGY | 0 | 1033 | 3180 | 0 | 1148 | 996 | 0 | 999 | 979 | 995 | 2061 | 981 | 998 | 0 |
| ESP | 1003 | 988 | 997 | 999 | 999 | 0 | 0 | 0 | 0 | 0 | 1025 | 999 | 1000 | 0 |
| EST | 0 | 0 | 1005 | 996 | 1010 | 994 | 991 | 991 | 995 | 997 | 1075 | 996 | 1017 | 0 |
| ETH | 0 | 0 | 0 | 0 | 994 | 999 | 988 | 985 | 993 | 996 | 2168 | 997 | 0 | 0 |
| FIN | 0 | 995 | 992 | 994 | 747 | 997 | 999 | 0 | 0 | 0 | 1025 | 1000 | 1005 | 0 |
| FRA | 975 | 995 | 983 | 984 | 734 | 989 | 998 | 0 | 0 | 0 | 1023 | 992 | 995 | 0 |
| GAB | 0 | 0 | 0 | 995 | 1006 | 1004 | 985 | 979 | 974 | 953 | 1060 | 0 | 1968 | 0 |
| GBR | 971 | 973 | 2507 | 0 | 739 | 1000 | 1000 | 0 | 0 | 0 | 1021 | 994 | 998 | 0 |
| GEO | 979 | 988 | 997 | 995 | 0 | 993 | 996 | 998 | 1000 | 991 | 1067 | 996 | 994 | 0 |
| GHA | 988 | 999 | 998 | 0 | 1003 | 981 | 986 | 975 | 947 | 985 | 1000 | 892 | 993 | 0 |
| GIN | 0 | 0 | 993 | 999 | 1008 | 1000 | 996 | 994 | 899 | 894 | 1082 | 0 | 1954 | 0 |
| GMB | 0 | 0 | 0 | 0 | 0 | 0 | 0 | 0 | 977 | 959 | 1101 | 0 | 0 | 0 |
| GRC | 994 | 992 | 997 | 998 | 998 | 999 | 1000 | 1000 | 999 | 995 | 1079 | 1001 | 1003 | 0 |
| GTM | 1004 | 997 | 983 | 979 | 988 | 960 | 917 | 940 | 971 | 948 | 1060 | 0 | 0 | 0 |
| HKG | 0 | 729 | 0 | 0 | 0 | 815 | 0 | 880 | 0 | 0 | 0 | 1004 | 971 | 0 |
| HND | 990 | 992 | 991 | 997 | 978 | 965 | 988 | 961 | 982 | 967 | 963 | 0 | 972 | 0 |
| HRV | 0 | 998 | 1001 | 940 | 981 | 991 | 994 | 974 | 1000 | 986 | 1077 | 988 | 991 | 0 |
| HTI | 0 | 503 | 502 | 503 | 504 | 498 | 503 | 501 | 499 | 485 | 0 | 0 | 0 | 0 |
| HUN | 0 | 1002 | 1007 | 0 | 0 | 998 | 995 | 991 | 999 | 995 | 1074 | 994 | 993 | 0 |
| IDN | 1080 | 1071 | 986 | 998 | 0 | 989 | 972 | 968 | 972 | 985 | 2118 | 0 | 2104 | 0 |
| IND | 2886 | 5958 | 3516 | 7465 | 5430 | 2969 | 2990 | 2998 | 2941 | 2962 | 0 | 6504 | 6009 | 0 |
| IRL | 476 | 958 | 963 | 985 | 979 | 993 | 995 | 0 | 0 | 0 | 1007 | 987 | 994 | 0 |
| IRN | 0 | 0 | 0 | 2481 | 989 | 992 | 990 | 987 | 0 | 0 | 0 | 1001 | 1004 | 0 |
| IRQ | 0 | 961 | 1978 | 0 | 0 | 997 | 977 | 998 | 0 | 0 | 2092 | 982 | 1002 | 0 |
| ISL | 0 | 0 | 0 | 993 | 494 | 0 | 0 | 588 | 0 | 0 | 0 | 495 | 496 | 0 |
| ISR | 996 | 990 | 993 | 973 | 996 | 986 | 994 | 994 | 1000 | 1007 | 0 | 2136 | 998 | 0 |
| ITA | 978 | 966 | 917 | 985 | 993 | 996 | 1000 | 0 | 0 | 0 | 1025 | 1000 | 1000 | 0 |
| JAM | 0 | 0 | 491 | 0 | 492 | 491 | 0 | 0 | 492 | 0 | 0 | 490 | 983 | 0 |
| JOR | 0 | 996 | 1994 | 0 | 996 | 997 | 998 | 996 | 1001 | 0 | 0 | 0 | 995 | 0 |
| JPN | 1000 | 998 | 999 | 0 | 994 | 993 | 986 | 993 | 0 | 0 | 0 | 1012 | 1004 | 0 |
| KAZ | 969 | 961 | 976 | 964 | 982 | 954 | 990 | 979 | 998 | 985 | 1056 | 892 | 983 | 0 |
| KEN | 0 | 999 | 0 | 999 | 988 | 998 | 999 | 994 | 994 | 980 | 985 | 996 | 995 | 0 |
| KGZ | 996 | 996 | 995 | 995 | 963 | 983 | 989 | 995 | 996 | 980 | 1056 | 978 | 995 | 0 |
| KHM | 0 | 1000 | 1000 | 995 | 0 | 991 | 985 | 974 | 1551 | 974 | 924 | 992 | 941 | 0 |
| KOR | 979 | 969 | 973 | 0 | 975 | 986 | 986 | 985 | 0 | 0 | 0 | 994 | 991 | 0 |
| KWT | 0 | 0 | 0 | 0 | 1008 | 0 | 1975 | 987 | 0 | 0 | 0 | 0 | 0 | 0 |
| LAO | 0 | 0 | 0 | 0 | 0 | 0 | 0 | 0 | 810 | 2135 | 846 | 922 | 836 | 0 |
| LBN | 0 | 0 | 1997 | 0 | 997 | 997 | 998 | 999 | 1000 | 997 | 0 | 2085 | 994 | 0 |
| LBR | 0 | 0 | 0 | 0 | 0 | 0 | 0 | 959 | 982 | 979 | 972 | 0 | 0 | 0 |
| LBY | 0 | 0 | 0 | 0 | 0 | 0 | 997 | 984 | 0 | 0 | 0 | 0 | 0 | 0 |
| LKA | 0 | 1015 | 994 | 982 | 1010 | 1045 | 1052 | 0 | 1067 | 1081 | 1061 | 994 | 995 | 0 |
| LSO | 0 | 0 | 998 | 0 | 0 | 0 | 0 | 983 | 959 | 0 | 969 | 0 | 0 | 0 |
| LTU | 490 | 970 | 980 | 987 | 965 | 980 | 973 | 964 | 989 | 884 | 957 | 976 | 992 | 0 |
| LUX | 0 | 995 | 986 | 987 | 494 | 994 | 1000 | 0 | 0 | 0 | 1024 | 0 | 0 | 0 |
| LVA | 510 | 0 | 1001 | 993 | 986 | 988 | 982 | 998 | 988 | 992 | 1064 | 993 | 1015 | 0 |
| MAR | 0 | 0 | 0 | 0 | 993 | 0 | 1050 | 966 | 957 | 859 | 925 | 972 | 962 | 0 |
| MDA | 979 | 988 | 977 | 963 | 983 | 984 | 970 | 988 | 0 | 992 | 1051 | 991 | 980 | 0 |
| MDG | 0 | 0 | 0 | 999 | 1007 | 1004 | 998 | 999 | 958 | 957 | 993 | 0 | 0 | 0 |
| MEX | 995 | 997 | 997 | 988 | 988 | 1016 | 1029 | 1000 | 989 | 1025 | 979 | 993 | 994 | 0 |
| MKD | 1004 | 992 | 966 | 1011 | 990 | 995 | 1005 | 1022 | 1004 | 1001 | 1069 | 993 | 1019 | 0 |
| MLI | 999 | 1000 | 1000 | 999 | 999 | 1000 | 976 | 961 | 946 | 930 | 1100 | 977 | 982 | 0 |
| MLT | 0 | 0 | 0 | 0 | 0 | 1007 | 991 | 0 | 0 | 0 | 1023 | 999 | 993 | 0 |
| MMR | 0 | 0 | 0 | 1016 | 1020 | 1020 | 1017 | 1017 | 1519 | 967 | 1076 | 996 | 999 | 0 |
| MNE | 0 | 0 | 992 | 995 | 992 | 1000 | 998 | 1000 | 997 | 995 | 1076 | 990 | 0 | 0 |
| MNG | 0 | 0 | 995 | 993 | 994 | 995 | 995 | 992 | 994 | 995 | 1066 | 998 | 997 | 0 |
| MOZ | 0 | 0 | 0 | 0 | 0 | 0 | 967 | 0 | 945 | 946 | 966 | 0 | 901 | 0 |
| MRT | 0 | 0 | 0 | 0 | 0 | 0 | 0 | 0 | 0 | 0 | 1075 | 0 | 0 | 0 |
| MUS | 0 | 0 | 995 | 0 | 0 | 997 | 0 | 995 | 0 | 0 | 0 | 994 | 987 | 0 |
| MWI | 999 | 0 | 1000 | 998 | 1000 | 1000 | 990 | 998 | 992 | 975 | 961 | 0 | 995 | 0 |
| MYS | 1002 | 998 | 965 | 998 | 988 | 999 | 1001 | 0 | 0 | 987 | 1027 | 0 | 1974 | 0 |
| NAM | 0 | 0 | 0 | 0 | 0 | 984 | 0 | 0 | 980 | 985 | 992 | 979 | 995 | 0 |
| NER | 993 | 1000 | 1000 | 1000 | 1008 | 1003 | 997 | 983 | 948 | 0 | 949 | 0 | 0 | 0 |
| NGA | 992 | 1000 | 0 | 1973 | 0 | 0 | 997 | 967 | 958 | 952 | 2914 | 948 | 988 | 0 |
| NIC | 1004 | 989 | 982 | 974 | 945 | 951 | 945 | 942 | 966 | 959 | 1053 | 993 | 968 | 0 |
| NLD | 0 | 991 | 971 | 986 | 743 | 998 | 1002 | 0 | 0 | 0 | 1027 | 1003 | 997 | 0 |
| NOR | 0 | 0 | 0 | 994 | 0 | 998 | 1000 | 0 | 0 | 0 | 0 | 1000 | 1007 | 0 |
| NPL | 996 | 0 | 973 | 908 | 1022 | 965 | 946 | 941 | 968 | 980 | 1954 | 997 | 986 | 0 |
| NZL | 0 | 737 | 988 | 995 | 496 | 986 | 992 | 986 | 0 | 0 | 0 | 975 | 969 | 0 |
| PAK | 1142 | 1022 | 1000 | 999 | 999 | 1000 | 1000 | 1000 | 1598 | 992 | 0 | 1086 | 2000 | 0 |
| PAN | 1013 | 998 | 997 | 999 | 1000 | 988 | 992 | 987 | 974 | 978 | 1069 | 0 | 983 | 0 |
| PER | 988 | 994 | 989 | 993 | 997 | 977 | 972 | 977 | 984 | 982 | 984 | 0 | 1994 | 0 |
| PHL | 994 | 994 | 989 | 1999 | 1000 | 1000 | 1000 | 1000 | 994 | 998 | 2085 | 996 | 998 | 0 |
| POL | 0 | 1905 | 1009 | 981 | 978 | 981 | 984 | 980 | 982 | 990 | 1065 | 975 | 979 | 0 |
| PRI | 0 | 0 | 0 | 0 | 0 | 496 | 0 | 0 | 0 | 0 | 0 | 0 | 0 | 0 |
| PRT | 0 | 1964 | 969 | 962 | 979 | 1005 | 1010 | 0 | 0 | 0 | 1021 | 993 | 995 | 0 |
| PRY | 987 | 984 | 987 | 983 | 994 | 998 | 995 | 998 | 0 | 1985 | 1069 | 999 | 985 | 0 |
| PSE | 0 | 997 | 1986 | 0 | 996 | 994 | 1000 | 996 | 999 | 995 | 1086 | 0 | 0 | 0 |
| QAT | 0 | 0 | 0 | 955 | 0 | 0 | 0 | 0 | 0 | 0 | 0 | 0 | 0 | 0 |
| ROU | 0 | 985 | 995 | 983 | 990 | 980 | 985 | 992 | 979 | 986 | 1053 | 0 | 1954 | 0 |
| RUS | 1965 | 3875 | 1961 | 2911 | 1971 | 1962 | 1968 | 1940 | 1953 | 1951 | 0 | 4940 | 1985 | 0 |
| RWA | 1000 | 0 | 996 | 1000 | 1000 | 1000 | 998 | 995 | 983 | 986 | 979 | 0 | 0 | 0 |
| SAU | 0 | 1020 | 2013 | 0 | 0 | 1010 | 1008 | 961 | 0 | 0 | 0 | 1038 | 1005 | 0 |
| SDN | 0 | 911 | 1996 | 1000 | 0 | 987 | 0 | 0 | 0 | 0 | 0 | 0 | 0 | 0 |
| SEN | 1000 | 1000 | 996 | 994 | 1000 | 1000 | 995 | 975 | 954 | 939 | 965 | 970 | 975 | 0 |
| SGP | 1005 | 1001 | 999 | 0 | 999 | 0 | 990 | 998 | 993 | 999 | 1037 | 0 | 992 | 0 |
| SLE | 0 | 0 | 0 | 0 | 0 | 0 | 0 | 954 | 969 | 965 | 1100 | 0 | 0 | 0 |
| SLV | 1004 | 993 | 980 | 993 | 997 | 978 | 961 | 942 | 977 | 974 | 1061 | 990 | 941 | 0 |
| SOM | 0 | 0 | 0 | 0 | 0 | 987 | 999 | 1183 | 0 | 0 | 0 | 0 | 0 | 0 |
| SRB | 0 | 990 | 990 | 1017 | 1028 | 993 | 994 | 998 | 993 | 983 | 1074 | 988 | 993 | 0 |
| SSD | 0 | 0 | 0 | 0 | 0 | 953 | 967 | 985 | 965 | 0 | 0 | 0 | 0 | 0 |
| SUR | 0 | 0 | 0 | 485 | 0 | 0 | 0 | 0 | 0 | 0 | 0 | 0 | 0 | 0 |
| SVK | 0 | 993 | 1000 | 1001 | 997 | 993 | 988 | 998 | 996 | 995 | 1052 | 991 | 998 | 0 |
| SVN | 498 | 980 | 984 | 991 | 994 | 1012 | 997 | 0 | 0 | 0 | 1010 | 987 | 999 | 0 |
| SWE | 0 | 998 | 990 | 994 | 747 | 997 | 1000 | 0 | 0 | 0 | 1023 | 996 | 1010 | 0 |
| SWZ | 0 | 0 | 1000 | 0 | 0 | 0 | 0 | 0 | 0 | 984 | 1079 | 0 | 0 | 0 |
| SYR | 0 | 0 | 1011 | 0 | 988 | 0 | 963 | 0 | 0 | 0 | 0 | 0 | 0 | 0 |
| TCD | 1000 | 1000 | 996 | 1000 | 998 | 1000 | 995 | 986 | 950 | 882 | 1075 | 0 | 0 | 0 |
| TGO | 0 | 0 | 999 | 0 | 0 | 995 | 983 | 987 | 960 | 963 | 1109 | 0 | 979 | 0 |
| THA | 1016 | 998 | 1000 | 1995 | 998 | 1000 | 0 | 962 | 952 | 964 | 1953 | 994 | 991 | 0 |
| TJK | 990 | 982 | 994 | 995 | 999 | 996 | 999 | 994 | 993 | 0 | 0 | 0 | 994 | 0 |
| TKM | 0 | 0 | 999 | 0 | 0 | 0 | 0 | 0 | 0 | 0 | 0 | 0 | 0 | 0 |
| TTO | 0 | 0 | 0 | 0 | 0 | 0 | 0 | 0 | 494 | 0 | 0 | 0 | 0 | 0 |
| TUN | 0 | 0 | 2026 | 0 | 1049 | 1048 | 994 | 993 | 997 | 972 | 972 | 996 | 984 | 0 |
| TUR | 986 | 995 | 1000 | 1981 | 997 | 990 | 990 | 995 | 993 | 993 | 2026 | 988 | 991 | 0 |
| TWN | 0 | 983 | 971 | 988 | 991 | 990 | 989 | 987 | 0 | 0 | 0 | 995 | 992 | 0 |
| TZA | 998 | 1000 | 999 | 1000 | 1007 | 999 | 1000 | 997 | 995 | 987 | 976 | 988 | 972 | 0 |
| UGA | 1000 | 1000 | 1000 | 1000 | 1000 | 985 | 972 | 992 | 925 | 975 | 977 | 997 | 993 | 0 |
| UKR | 1051 | 993 | 980 | 970 | 977 | 0 | 967 | 990 | 988 | 973 | 1066 | 988 | 978 | 0 |
| URY | 975 | 964 | 971 | 994 | 997 | 991 | 995 | 988 | 991 | 997 | 0 | 2069 | 995 | 0 |
| USA | 1001 | 986 | 557 | 0 | 497 | 996 | 0 | 525 | 0 | 0 | 0 | 1002 | 997 | 0 |
| UZB | 958 | 972 | 990 | 992 | 985 | 983 | 991 | 976 | 992 | 996 | 1069 | 989 | 987 | 0 |
| VEN | 855 | 984 | 989 | 987 | 983 | 992 | 994 | 995 | 995 | 0 | 0 | 0 | 0 | 0 |
| VNM | 996 | 0 | 903 | 949 | 989 | 943 | 947 | 1021 | 0 | 969 | 1960 | 0 | 1947 | 0 |
| XKX | 0 | 1011 | 1042 | 1012 | 995 | 999 | 978 | 994 | 991 | 995 | 1087 | 998 | 998 | 0 |
| XNC | 0 | 0 | 0 | 0 | 494 | 995 | 983 | 0 | 0 | 0 | 0 | 0 | 0 | 0 |
| XNK | 0 | 0 | 0 | 0 | 995 | 0 | 0 | 0 | 0 | 0 | 0 | 0 | 0 | 0 |
| XSR | 0 | 1000 | 2000 | 1000 | 0 | 0 | 0 | 0 | 0 | 0 | 0 | 0 | 0 | 0 |
| YEM | 0 | 998 | 1972 | 0 | 996 | 996 | 992 | 997 | 997 | 991 | 1112 | 0 | 0 | 0 |
| ZAF | 0 | 1000 | 1000 | 1997 | 1000 | 998 | 999 | 995 | 992 | 980 | 1041 | 1007 | 1030 | 0 |
| ZMB | 994 | 0 | 997 | 997 | 1000 | 996 | 994 | 962 | 982 | 964 | 973 | 1019 | 968 | 0 |
| ZWE | 0 | 1000 | 991 | 994 | 997 | 989 | 994 | 981 | 983 | 990 | 0 | 0 | 0 | 0 |
| Note: 0 means that there is no Gallup World Poll survey in a certain country in a specific year. | | | | | | | | | | | | | | |

**None: Table S2** (including **Tables S1.1**, **S1.2**, **S1.3**, **S1.4**, and **S1.5**) summarizes the coefficients of each equation listed in **Equation 2**. **Equation 2** includes 26 equations, but two equations are not displayed because two variables, satisfaction with educational systems and the quality of air, are affected by only unobserved variables.

| **Table S2.1: Results of DAG of the Relationships among Variables** | | | | | |
| --- | --- | --- | --- | --- | --- |
|  | Dependent variable: | | | | |
|  | TWR | LGH | ENJ | INT | WLR |
|  | (1) | (2) | (3) | (4) | (5) |
| AGE | 0.005^***^ | -0.013^***^ | -0.009^***^ | -0.013^***^ | -0.004^***^ |
|  | (0.0001) | (0.0001) | (0.0001) | (0.0001) | (0.0001) |
| Constant | 1.628^***^ | 1.440^***^ | 1.157^***^ | 0.615^***^ | 0.866^***^ |
|  | (0.006) | (0.005) | (0.004) | (0.004) | (0.004) |
| Observations | 1,582,367 | 1,582,367 | 1,582,367 | 1,582,367 | 1,582,367 |
| Log Likelihood | -634,868.700 | -948,922.600 | -977,341.400 | -1,085,406.000 | -1,001,614.000 |
| Akaike Inf. Crit. | 1,269,741.000 | 1,897,849.000 | 1,954,687.000 | 2,170,817.000 | 2,003,231.000 |
| Note: | ^*^p<0.1 ^**^p<0.05 ^***^p<0.01 | | | | |

| **Table S2.2: Results of DAG of the Relationships among Variables** | | | | | |
| --- | --- | --- | --- | --- | --- |
|  | Dependent variable: | | | | |
|  | PHP | STR | SDN | ANG | WRR |
|  | (1) | (2) | (3) | (4) | (5) |
| AGE | 0.009^***^ | -0.007^***^ | 0.012^***^ | -0.013^***^ | 0.009^***^ |
|  | (0.0001) | (0.0001) | (0.0001) | (0.0001) | (0.0001) |
| HPD | 1.323^***^ |  |  |  |  |
|  | (0.004) |  |  |  |  |
| PHP |  | 1.032^***^ |  | 1.024^***^ |  |
|  |  | (0.004) |  | (0.004) |  |
| NOF |  |  | 0.775^***^ |  | 0.784^***^ |
|  |  |  | (0.004) |  | (0.003) |
| NOH |  |  | 0.357^***^ |  |  |
|  |  |  | (0.005) |  |  |
| Constant | -1.582^***^ | -0.785^***^ | -2.062^***^ | -1.250^***^ | -1.110^***^ |
|  | (0.005) | (0.004) | (0.006) | (0.005) | (0.005) |
| Observations | 1,582,367 | 1,582,367 | 1,582,367 | 1,582,367 | 1,582,367 |
| Log Likelihood | -906,251.700 | -960,505.000 | -829,780.000 | -760,464.300 | -1,024,346.000 |
| Akaike Inf. Crit. | 1,812,509.000 | 1,921,016.000 | 1,659,568.000 | 1,520,935.000 | 2,048,698.000 |
| Note: | ^*^p<0.1 ^**^p<0.05 ^***^p<0.01 | | | | |

| **Table S2.3: Results of DAG of the Relationships among Variables** | | | | | |
| --- | --- | --- | --- | --- | --- |
|  | Dependent variable: | | | | |
|  | UNE | HPD | NOF | NOH | SPT |
|  | (1) | (2) | (3) | (4) | (5) |
| AGE | -0.019^***^ | 0.038^***^ |  |  |  |
|  | (0.0002) | (0.0001) |  |  |  |
| GEN | -0.158^***^ |  |  |  |  |
|  | (0.006) |  |  |  |  |
| INC(log) |  |  | -0.394^***^ | -0.242^***^ | 0.083^***^ |
|  |  |  | (0.001) | (0.001) | (0.001) |
| Constant | -1.645^***^ | -2.777^***^ | 2.332^***^ | 0.740^***^ | -0.321^***^ |
|  | (0.008) | (0.005) | (0.008) | (0.007) | (0.006) |
| Observations | 1,582,367 | 1,582,367 | 1,582,367 | 1,582,367 | 1,582,367 |
| Log Likelihood | -432,781.700 | -823,855.600 | -925,143.000 | -859,315.200 | -1,071,928.000 |
| Akaike Inf. Crit. | 865,569.500 | 1,647,715.000 | 1,850,290.000 | 1,718,634.000 | 2,143,861.000 |
| Note: | ^*^p<0.1 ^**^p<0.05 ^***^p<0.01 | | | | |

| **Table S2.4: Results of DAG of the Relationships among Variables** | | | | | |
| --- | --- | --- | --- | --- | --- |
|  | Dependent variable: | | | | |
|  | SRH | SWQ | SHC | SAH | STC |
|  | (1) | (2) | (3) | (4) | (5) |
| INC(log) | 0.136^***^ | 0.138^***^ | 0.143^***^ | 0.019^***^ |  |
|  | (0.001) | (0.001) | (0.001) | (0.001) |  |
| NOH |  |  |  | -0.339^***^ |  |
|  |  |  |  | (0.004) |  |
| SPT |  |  |  |  | 0.558^***^ |
|  |  |  |  |  | (0.004) |
| SRH |  |  |  |  | 0.443^***^ |
|  |  |  |  |  | (0.005) |
| SAQ |  |  |  |  | 0.384^***^ |
|  |  |  |  |  | (0.005) |
| SWQ |  |  |  |  | 0.426^***^ |
|  |  |  |  |  | (0.004) |
| SAH |  |  |  |  | 0.429^***^ |
|  |  |  |  |  | (0.004) |
| SES |  |  |  |  | 0.453^***^ |
|  |  |  |  |  | (0.004) |
| SHC |  |  |  |  | 0.478^***^ |
|  |  |  |  |  | (0.005) |
| Constant | -0.919^***^ | -0.282^***^ | -0.882^***^ | -0.142^***^ | -0.454^***^ |
|  | (0.007) | (0.007) | (0.007) | (0.007) | (0.004) |
| Observations | 1,582,367 | 1,582,367 | 1,582,367 | 1,582,367 | 1,582,367 |
| Log Likelihood | -1,079,494.000 | -974,318.100 | -1,072,432.000 | -1,090,406.000 | -745,043.900 |
| Akaike Inf. Crit. | 2,158,991.000 | 1,948,640.000 | 2,144,868.000 | 2,180,817.000 | 1,490,104.000 |
| Note: | ^*^p<0.1 ^**^p<0.05 ^***^p<0.01 | | | | |

| **Table S2.5: Results of DAG of the Relationships among Variables** | | | | |
| --- | --- | --- | --- | --- |
|  | Dependent variable: | | | |
|  | RTL | MAC | INC(log) | LE |
|  | logistic | logistic | OLS | OLS |
|  | (1) | (2) | (3) | (4) |
| SPT | 0.282^***^ | -0.181^***^ |  | 0.001 |
|  | (0.004) | (0.005) |  | (0.004) |
| SRH | 0.195^***^ | -0.101^***^ |  | 0.120^***^ |
|  | (0.004) | (0.005) |  | (0.004) |
| SAQ | 0.301^***^ | -0.110^***^ |  | -0.132^***^ |
|  | (0.004) | (0.005) |  | (0.004) |
| SWQ | 0.251^***^ | -0.223^***^ |  | 0.173^***^ |
|  | (0.004) | (0.005) |  | (0.004) |
| SAH | 0.267^***^ | -0.093^***^ |  | 0.106^***^ |
|  | (0.004) | (0.005) |  | (0.004) |
| SES | 0.278^***^ | -0.157^***^ |  | 0.093^***^ |
|  | (0.004) | (0.005) |  | (0.004) |
| SHC | 0.300^***^ | -0.119^***^ |  | 0.212^***^ |
|  | (0.004) | (0.005) |  | (0.004) |
| UNE |  |  | -0.573^***^ | -0.097^***^ |
|  |  |  | (0.006) | (0.006) |
| AGE |  |  | 0.026^***^ |  |
|  |  |  | (0.0001) |  |
| GEN |  |  | -0.065^***^ |  |
|  |  |  | (0.003) |  |
| HPD |  |  | -0.610^***^ | -0.359^***^ |
|  |  |  | (0.004) | (0.004) |
| WLR |  |  |  | 0.106^***^ |
|  |  |  |  | (0.004) |
| PHP |  |  | -0.432^***^ | -0.059^***^ |
|  |  |  | (0.003) | (0.004) |
| COU |  |  | -0.002^***^ |  |
|  |  |  | (0.00003) |  |
| WRR |  |  |  | -0.167^***^ |
|  |  |  |  | (0.004) |
| SDN |  |  |  | -0.206^***^ |
|  |  |  |  | (0.005) |
| TWR |  |  |  | 0.172^***^ |
|  |  |  |  | (0.005) |
| LGH |  |  |  | 0.220^***^ |
|  |  |  |  | (0.004) |
| INT |  |  |  | 0.364^***^ |
|  |  |  |  | (0.004) |
| ENJ |  |  |  | 0.329^***^ |
|  |  |  |  | (0.004) |
| STR |  |  |  | -0.019^***^ |
|  |  |  |  | (0.004) |
| ANG |  |  |  | -0.037^***^ |
|  |  |  |  | (0.005) |
| NOF |  |  |  | -0.708^***^ |
|  |  |  |  | (0.004) |
| NOH |  |  |  | -0.195^***^ |
|  |  |  |  | (0.004) |
| STC |  |  |  | 0.284^***^ |
|  |  |  |  | (0.005) |
| MAC |  |  |  | 0.106^***^ |
|  |  |  |  | (0.005) |
| RTL |  |  |  | 0.297^***^ |
|  |  |  |  | (0.004) |
| INC(log) |  |  |  | 0.246^***^ |
|  |  |  |  | (0.001) |
| Constant | -0.244^***^ | -1.001^***^ | 7.157^***^ | 2.533^***^ |
|  | (0.004) | (0.005) | (0.005) | (0.010) |
| Observations | 1,582,367 | 1,582,367 | 1,582,367 | 1,582,367 |
| R^2^ |  |  | 0.077 | 0.214 |
| Adjusted R^2^ |  |  | 0.077 | 0.214 |
| Log Likelihood | -924,705.700 | -716,586.300 |  |  |
| Akaike Inf. Crit. | 1,849,427.000 | 1,433,189.000 |  |  |
| Residual Std. Error |  |  | 1.932 (df = 1582360) | 2.144 (df = 1582341) |
| F Statistic |  |  | 21,976.480^***^ (df = 6; 1582360) | 17,233.480^***^ (df = 25; 1582341) |
| Note: | ^*^p<0.1 ^**^p<0.05 ^***^p<0.01 | | | |
